# Catalytic properties of the integral membrane RAS protease Rce1

**DOI:** 10.1016/j.jbc.2026.113525

**Published:** 2026-09-08

**Authors:** David J. Van Dongen, Abbie M. Collette, Melinda M. Diver, Stephen B. Long

**Affiliations:** 1 Structural Biology Program, Sloan Kettering Institute, Memorial Sloan Kettering Cancer Center, New York, New York, USA; 2 Louis V. Gerstner Jr. Graduate School of Biomedical Sciences, Memorial Sloan Kettering Cancer Center, New York, New York, USA; 3 Graduate Program in Biochemistry and Structural Biology, Cell and Developmental Biology, and Molecular Biology, Weill Cornell Medicine Graduate School of Medical Sciences, New York, New York, USA

**Keywords:** Ras protein, intramembrane proteolysis, CaaX protein prenylation, enzyme purification, enzyme kinetics, membrane enzyme

## Abstract

The integral membrane protease Rce1 cleaves prenylated CaaX proteins, including all RAS GTPases, as part of their posttranslational maturation. This cleavage, which removes the -aaX residues, is necessary for the proper cellular location and signaling properties of RAS and its oncogenic forms. Poor biochemical stabilities of yeast and mammalian Rce1 have hindered the purification of Rce1 in active form and limited studies addressing its catalytic properties. Here, we identify that *Drosophila melanogaster* Rce1 has good biochemical stability and is catalytically active when purified. The purified protein recapitulates known properties of Rce1, including specificity for prenylated CaaX substrates. Like other integral membrane proteases, the enzyme has a low turnover number (*k*_cat_), of approximately 20 h^−1^. Pre-steady-state kinetic analysis reveals that product release is the rate-limiting step. Our data indicate that Rce1 is inhibited by nanomolar levels of Zn^2+^, but that metals are not involved in catalysis. The data suggest that Zn^2+^ is a competitive inhibitor with respect to the substrate. The signaling lipid PA also inhibits the enzyme *in vitro*, raising the possibility that the cellular activity of Rce1 may be modulated by lipids and/or Zn^2+^. These studies combined with structural predictions and prior investigation lead to the hypothesis that the active site contains a catalytic Glu-His-His triad of amino acids that coordinate the substrate and form the inhibitory Zn^2+^ site. This work enhances the understanding of the enzyme and gives insight into means for Rce1 inhibition.

Many membrane-associated proteins undergo posttranslational modifications that affect their subcellular localization and function. The more than 200 mammalian proteins that contain a C-terminal CaaX sequence motif, which consists of cysteine (C) followed by two aliphatic amino acids (aa) and a terminal amino acid (X), undergo three sequential steps of enzymatic modification that enhance membrane affinity and/or specific protein–protein interactions (1, 2, 3, 4, 5, 6). The cohort of thus modified proteins includes all RAS GTPases for which each step has been shown to be necessary for proper localization and signaling function (4, 6, 7, 8). In the first enzymatic step, the cysteine residue is prenylated with either a farnesyl (C15) or geranylgeranyl (C20) prenyl lipid by the enzyme FTase or GGTase-I, respectively (Figure 1). Next, the RAS-converting enzyme (Rce1) protease cleaves the peptide bond adjacent to the prenylcysteine, thereby removing the -aaX residues (9, 10, 11). Finally, isoprenylcysteine carboxylmethyltransferase (ICMT) catalyzes methylation of the C-terminal carboxylate of the prenylcysteine residue. Both Rce1 and ICMT are integral membrane proteins located in the endoplasmic reticulum (ER) membrane and both enzymes require their respective substrates to contain a prenyl group (9, 10, 12). While structural and functional studies of FTase, GGTase-I, and ICMT have yielded insights into their respective mechanisms, our understanding of the mechanisms of Rce1 has lagged, in part, because it has not been purified in catalytically active form (13, 14, 15).

**Figure 1:**
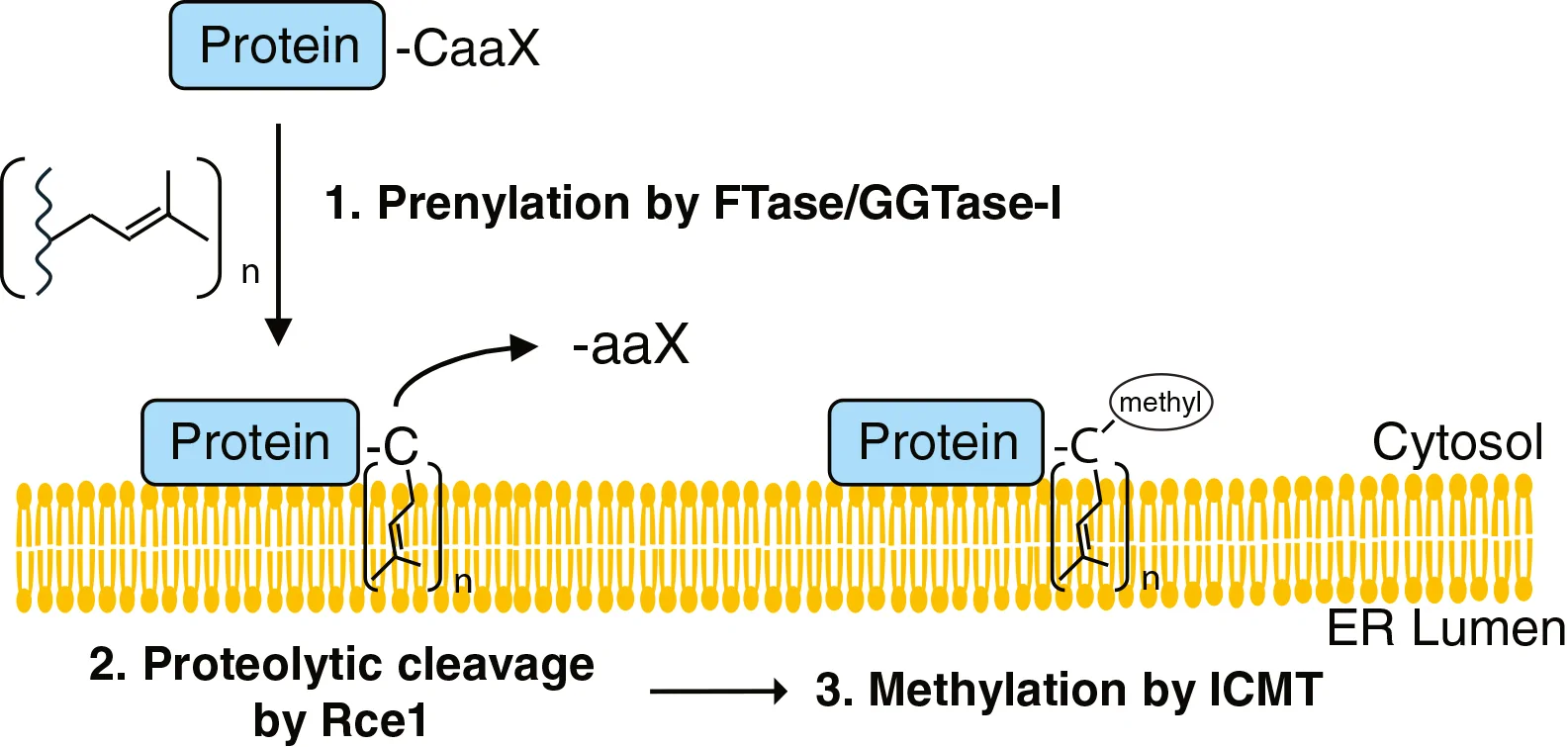
**Posttranslational processing of CaaX proteins.** Proteins containing a C-terminal CaaX amino acid sequence motif undergo three enzymatic steps: 1, prenylation, which comprises the attachment of a 15-carbon farnesyl (n = 3) or 20-carbon geranylgeranyl (n = 4) prenyl moiety to the cysteine (‘C’) residue of the CaaX motif by the cytosolic enzymes FTase or GGTase-I, respectively. 2, proteolysis of the prenylated CaaX motif between the prenylated cysteine and the ‘aaX’ residues by the protease Rce1, which is multipass transmembrane protein located in the ER membrane. 3, methylation of the newly formed C-terminal carboxylate of the prenylated cysteine by the enzyme ICMT, which is also a multipass transmembrane protein of the ER. Following processing, the more than 200 different CaaX proteins are trafficked to cellular destinations. The three-step process increases the hydrophobicity of the modified protein, facilitating its association with cellular membranes (often the plasma membrane, as in the case of RAS, but sometimes the nuclear membrane, as in the case of lamins) or stabilizing specific protein–protein interactions. ER, endoplasmic reticulum; ICMT, isoprenylcysteine carboxylmethyltransferase.

The critical role of membrane localization for the signaling of RAS proteins, which are among the most potent oncogenic drivers in cancer, has generated considerable interest in therapeutically targeting the CaaX processing enzymes for the treatment of various cancers (16, 17, 18, 19, 20, 21, 22). Although some clinical trials are ongoing (*e.g.* NCT06026410), the inhibition of FTase for RAS-driven cancers, which showed strong potential from preclinical experiments, have been ineffective in completed clinical trials, potentially due to compensation by GGTase-I (17, 23, 24). Rce1 and ICMT have been postulated to be more promising therapeutic targets, as there may be less compensatory enzymatic functionality *in vivo* (21, 25, 26). For example, data indicate that the prenylated CaaX motifs of RAS GTPases are exclusively cleaved by Rce1 in cells (21, 27, 28, 29, 30, 31).

Studies using cell membrane-derived microsomes that contain Rce1 have established properties of the enzyme’s substrate specificity, including the requirement for a prenyl lipid (9, 10, 32). However, the catalytic rate of Rce1 is not known because of an indeterminate amount of enzyme in the microsome preparations. The primary amino acid sequence of Rce1 has no discernible homology with other known proteases, suggesting that it has distinct mechanisms for substrate recognition and/or peptide bond cleavage. Various approaches have been used to investigate these mechanisms. Initial reports suggested that Rce1 was a cysteine protease based on inhibition by TPCK, a potent irreversible inhibitor of serine and cysteine proteases (33, 34). However, this mechanism was later ruled out when it was demonstrated that a cysteine-free mutant of *Saccharomyces cerevisiae* Rce1 (*Sc*Rce1) retained activity in cells (35).

Mutagenesis studies of *Sc*Rce1, human Rce1, and a distantly related archaeal protease (from *Methanococcus maripaludis)* identified a triad of conserved amino acids – E175, H208, and H261 (human Rce1 numbering) – that eliminate enzymatic activity when any is mutated (9, 29, 36, 37). Two hypotheses for the involvement of these amino acids in the mechanism have been put forth. Because histidine and glutamate residues can form the binding site for zinc ions, one hypothesis is that Rce1 is a zinc metalloprotease and these amino acids coordinate a catalytic zinc ion (29, 38). Supporting this hypothesis, exogenous zinc has been shown to inhibit Rce1, and this property, somewhat ironically, has been shown to correlate with zinc serving a catalytic function in other metalloproteases (9, 34, 39, 40, 41). However, most metal-chelating agents do not inhibit the enzyme, suggesting that zinc or other metals may not be involved in catalysis (29, 33, 36, 42). High (5 mM) concentrations of the metal-chelator 1,10-phenanthroline have been shown to inhibit Rce1, but data suggest that the inhibitory property of this compound is because it causes denaturation of the enzyme (36). Studies of the archaeal enzyme indicate that it does not contain zinc, but this enzyme shares only 13% sequence identity with human Rce1 (*Hs*Rce1), raising the possibility of a distinct mechanism (36). At present, it is unresolved whether eukaryotic Rce1 enzymes require zinc or some other metal for catalysis.

The other hypothesis is that Rce1 operates through a proteolytic mechanism distinct from other integral membrane enzymes that involves the conserved triad of E175, H208, and H261 residues (9, 29, 36, 37). Support for this mechanism comes from an X-ray structure of the archaeal protease (36). Although there are, as yet, no structures of substrate or product complexes that would further inform an understanding of its mechanism, the residues that may correspond to E175, H208, and H261 of human Rce1 are located in the presumed active site in the apo structure. In the structure, these residues coordinate a water molecule. Based on this structure, it was proposed that the glutamate residue activates a water molecule for nucleophilic attack and hydrolysis of the peptide bond. Such a mechanism would represent the only known intramembrane protease that uses a catalytic glutamate residue (43).

Archaeal organisms are not thought to possess prenylated proteins, suggesting that the native substrate of the *M. maripaludis* protease, which remains unknown, is not a prenylated peptide (44, 45). Several functional properties distinguish this protease from eukaryotic Rce1 enzymes, raising the possibility that their mechanisms differ. For example, the archaeal enzyme is not inhibited by zinc (36). Its substrate specificity is narrower: while eukaryotic Rce1 enzymes cleave farnesylated or geranylgeranylated peptides, the archaeal enzyme could not cleave geranylgeranylated ones *in vitro*. It also cleaves at an alternative position, preferring the bond between the two aliphatic amino acids (Ca-aX) over the canonical C-aaX site for the farnesylated peptides tested. These differences may limit the extent to which the archaeal enzyme serves as a model for eukaryotic Rce1. The mechanism of CaaX cleavage by Rce1 remains unresolved.

We reasoned that prior attempts to purify eukaryotic Rce1 enzymes in catalytically active form may have been unsuccessful because of poor biochemical stability of the protein in detergent-containing solutions. In this study, we screened numerous metazoan orthologs to identify those that had better biochemical stability. Rce1 from *Drosophila melanogaster* (*Dm*Rce1) stood out as a viable candidate and was purified. Purified *Dm*Rce1 is catalytically active in detergent-containing solutions and was used to characterize its enzymatic properties. Like the activity of Rce1 enzymes that have been evaluated using microsome preparations, purified *Dm*Rce1 exhibits specificity for CaaX peptides that are prenylated. We examined the effects of lipids, pH, and divalent cations, and identified conditions that stimulate or suppress enzyme activity, including potent inhibition by Zn^2+^. From metal analysis of the purified protein, however, our findings indicate that Rce1 is not a metalloenzyme. Leveraging the enzyme’s low turnover number, we used pre-steady-state kinetics to determine the rate-limiting step of the reaction.

## Results

### Purification of catalytically active Rce1

*Dm*Rce1 was selected for these studies for its robust expression when expressed in mammalian or yeast cells and good biochemical stability in detergent-containing solutions, which were evaluated using fluorescence-detection size exclusion chromatography (FSEC) analyses of numerous eukaryotic Rce1 orthologs (Experimental Procedures). *Dm*Rce1 shares approximately 38% amino acid sequence identity with human Rce1 and 30% identity with *S. cerevisiae* Rce1 (Fig. S1). The AlphaFold3 predicted three-dimensional structures of *Hs*Rce1 and *Dm*Rce1 have an RMSD of 0.7 Å for Cα atoms and the same overall fold, which suggests that the proteins are structurally analogous (Fig. S2) (46).

For large scale protein production, *Dm*Rce1 (hereafter referred to as Rce1 unless noted) was expressed in the yeast *Pichia pastoris* as a fusion protein with GFP with a cleavable linker between the two proteins. The recombinantly expressed protein was extracted from yeast membranes using the mild detergent tetradecylmaltoside (TDM) and purified by GFP-affinity chromatography, followed by removal of the GFP tag by proteolytic cleavage. The Rce1 protein was then further purified by size-exclusion chromatography (SEC) using a buffer containing another mild detergent, glyco-diosgenin (GDN) (Fig. 2*A*). The detergent exchange from TDM into GDN dramatically increased the catalytic activity of the protein (Fig. 2*C*). Because Rce1 was extracted from cellular membranes much more effectively by TDM than GDN, we used the TDM-to-GDN exchange method for protein purification and enzymatic studies.

**Figure 2:**
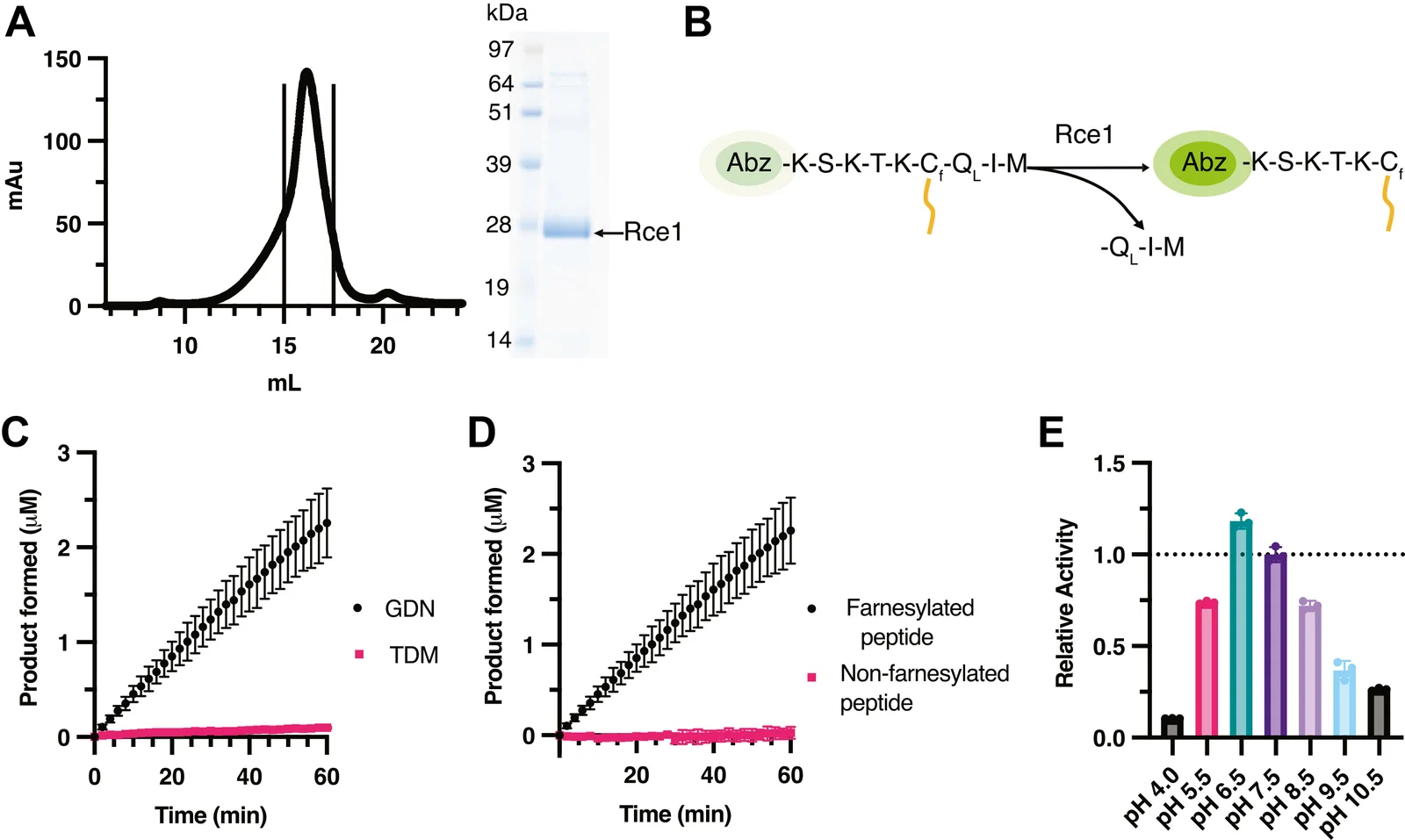
**Enzymatic activity of purified Rce1.***A*, size-exclusion chromatography (SEC) trace of purified *Dm*Rce1 in the detergent GDN (*left*). *Vertical lines* indicate the fraction used for enzymatic studies. Coomassie stained SDS-PAGE gel analysis of the purified protein, with molecular weight marker (*right*). *B*, schematic of the enzymatic activity assay, which uses a peptide substrate that mimics the C-terminal region of human KRAS-4B. Proteolytic cleavage of this internally quenched peptide by Rce1 liberates the quencher (dinitrophenyl lysine, Q_L_) and causes an increase of fluorescence in the 2-aminobenzoyl (Abz) group. A concentration of 20 μM peptide is used in all assays unless noted. *C*, activity of Rce1 in the detergents TDM and GDN. Protein prepared using TDM throughout purification (*red trace*) compared to protein that had been partially purified using TDM and then exchanged into GDN using SEC (*black trace*). The concentration of product formed is plotted as a function of time (n = 8 for GDN sample; n = 3 for TDM sample.). Fluorescence readings were converted into the concentration of product using peptide standards (Experimental Procedures). *D*, specificity of purified Rce1 for prenylated substrates. The enzymatic activity using peptides with or without a farnesyl group attached to the cysteine residue are compared (n = 8 for the farnesylated sample; n = 5 for the non-farnesylated sample). *E*, pH dependence of activity. The standard assay was performed for 1 h at the indicated pH values (n = 3 for each condition). Values are normalized to pH 7.5, and they are adjusted to account for small pH-dependent changes in the fluorescence of synthetic farnesylated product peptide (Fig. S4). Error bars represent SEM in all figures, unless noted. Rce1, RAS-converting enzyme; *Dm*Rce1, Rce1 from *Drosophila melanogaster*; GDN, glyco-diosgenin; TDM, tetradecylmaltoside.

The catalytic activity of purified Rce1 was evaluated using a previously described Rce1 activity assay in which an internally quenched fluorescent peptide is used as a substrate (Fig. 2*B*) (47, 48). This peptide mimics the nine C-terminal amino acids of the K-RAS4b protein and contains a farnesylcysteine residue in the CaaX motif. The peptide also includes a 2-aminobenzoyl (Abz) fluorophore at its N terminus and a dinitrophenyl lysine quencher moiety in the a_1_ position of the Ca_1_a_2_X motif. Following proteolytic cleavage between the farnesylcysteine and a_1_ residues, the quencher and fluorophore become separated, which allows the enzymatic activity of Rce1 to be measured from an increase in fluorescence. The assay allows the formation of product to be measured in real time by making periodic fluorescence measurements. The standard assay was performed at 25 °C using 300 nM Rce1 and 20 μM substrate peptide. The relatively high concentration of enzyme was used to achieve robust fluorescence signals. The amount of product formed was determined by converting the fluorescence signal into a molar quantity using standard curves generated from known quantities of synthetic peptides corresponding to the cleavage products (Experimental Procedures).

Purified Rce1 demonstrated robust activity, readily cleaving the farnesylated peptide, as indicated by a time-dependent increase in fluorescence (Fig. 2*D*). Activity was not observed using an analogous peptide without the farnesyl group, which indicates that the purified enzyme exhibits specificity for substrates that contain an isoprenoid lipid, as has been shown previously for microsomes containing other eukaryotic Rce1 orthologs (10, 32). The purified enzyme thereby recapitulates hallmark properties of Rce1 observed using cellular membranes.

Using the reconstituted system, we evaluated the pH optimum of the enzyme from pH 4.0 to pH 10.5. The enzyme displayed robust activity from pH 5.5 to pH 8.5, but it was less catalytically active at higher or lower pH (Fig. 2*E*). Maximal activity was observed at pH 6.5, but this was only marginally higher than that observed at pH 7.5. The pH 7.5 condition was chosen as the standard assay condition to more closely mimic physiological pH.

### Kinetic characterization of Rce1

We next sought to determine the kinetic parameters of the purified enzyme and of the enzyme in cellular membranes. For Rce1 in cellular membranes (microsomes), membranes from lysed *P. pastoris* cells that had been transformed with GFP-tagged *Dm*Rce1 were isolated, sonicated, and used in the activity assay (Experimental Procedures). Following methods analogous to those described previously for the kinetic analysis of ICMT in microsomes (49), the amount of Rce1 in the microsomes was quantified from the fluorescence of the attached GFP tag by comparing the amount of detergent-solubilized Rce1-GFP in the membranes to purified GFP standards using FSEC. To account for the endogenous Rce1 enzymatic activity of *P. pastoris* and other possible cellular effects, background levels of proteolytic activity were subtracted using membranes from untransformed cells (Fig. S5). Like the detergent purified protein, microsomal Rce1-GFP readily catalyzed cleavage of the farnesylated peptide (Fig. 3*A*). A plot of substrate concentration *versus* enzymatic rate revealed a relationship consistent with Michaelis-Menten behavior. Fitting the data to the standard Michaelis-Menten model yielded a turnover number (*k*_cat_) of 20.0 ± 3.2 h^−1^ and an apparent Michaelis constant (*K*_M,app_) of 56 ± 16 μM. We refer to *K*_M,app_ as an apparent value because the effective concentration of the substrate in the vicinity of the enzyme may depend on the partitioning of the substrate into micelles/membranes. Estimates of the *K*_M,app_ from *Sc*Rce1, *Hs*Rce1, and archaeal enzymes have yielded values of approximately 1.7 μM, 3.5 μM, and 19.7 μM, respectively (29, 36, 50). Previous studies of eukaryotic orthologs have not evaluated *k*_cat_ because the amount of Rce1 enzyme was unknown in those assays. While orders of magnitude lower than those of many water-soluble proteases, the measured *k*_cat_ is consistent with the low turnover numbers of other integral membrane proteases (51, 52, 53, 54).

**Figure 3:**
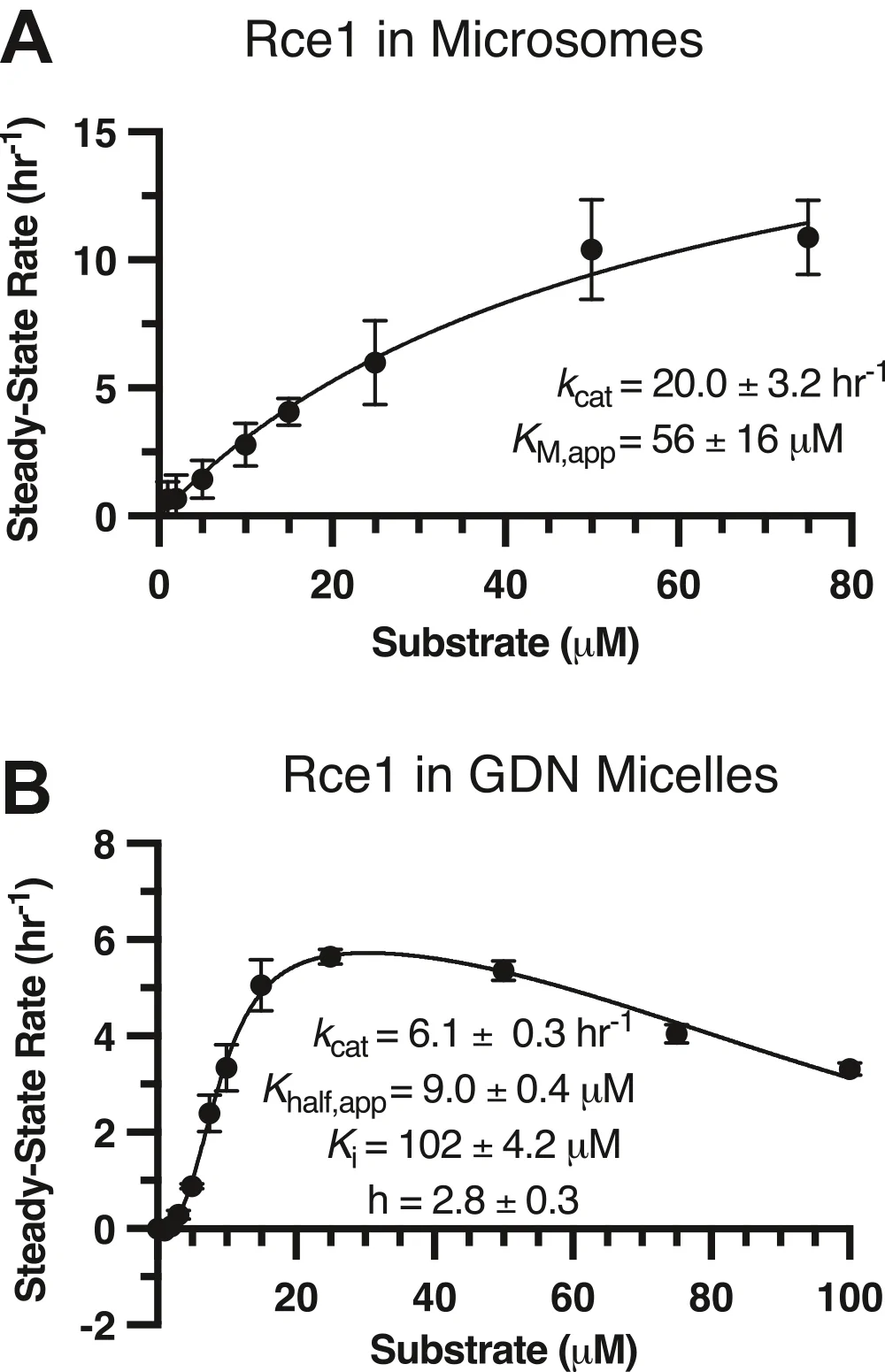
**Kinetic characterization of DmRce1.***A*, catalytic activity of unpurified *Dm*Rce1 in microsomes, shown as a plot of the steady-state rate as a function of substrate (farnesylated peptide) concentration. Steady-state rates were determined from linear regressions of the product formed *versus* time relationships. Microsomes were prepared from *Pichia pastoris* membranes containing heterologously expressed *Dm*Rce1 (Experimental Procedures). Background proteolytic activity was subtracted using control membranes from *Pichia pastoris* that do not express *Dm*Rce1 (*e.g.*Fig. S5). The *solid line* shows a Michaelis-Menten fit of the data. The derived kinetic parameters are indicated with S.E. of the fit. *B*, activity of purified *Dm*Rce1 (in GDN detergent) shown in the same manner as (*A*). The solid line represents a fit of the data to an allosteric sigmoidal kinetic model that takes substrate inhibition into consideration (Experimental Procedures). Derived parameters are indicated with S.E. of the fit. n = 3 for (*A*) and (*B*). *Dm*Rce1, Rce1 from *Drosophila melanogaster*; GDN, glyco-diosgenin.

A plot of substrate concentration *versus* enzymatic rate for purified Rce1 revealed a different relationship to that observed for Rce1 in microsomes (Fig. 3*B*). Following a steep rise of the enzymatic rate in the 0 to 10 μM substrate range, the rate gradually declines with higher substrate concentrations to approximately 60% of its maximal value with 100 μM substrate. The decline could be the result of substrate inhibition wherein higher concentrations of substrate inhibit the enzyme, possibly due to a second inhibitory binding site on the enzyme, or other effects due to detergent micelles (49, 55). The data can be fit with an allosteric sigmoidal model with substrate inhibition (Fig. 3*B*). Doing so yields a *k*_cat_ of approximately 6 h^−1^, an inhibitory constant, *K*_i_, of approximately 100 μM, and an apparent *K*_half_ (*K*_half,app_), representing the substrate concentration for half-maximal velocity, of approximately 9 μM. The few-fold higher apparent affinity of the purified protein for its substrate in comparison to microsomal preparations may reflect the smaller surface area of a micelle into which a hydrophobic substrate would presumably diffuse relative to a lipid membrane and would be analogous to differences that have been observed for other integral membrane proteases (52).

### Lipids affect the catalytic activity of purified Rce1 and alleviate substrate inhibition

We wondered if lipids might affect the activity of purified Rce1, since the enzymatic activity of membrane proteins is often influenced by lipids (56, 57, 58, 59). For example, the addition of solubilized lipids to detergent purified protein can create lipid-detergent micelles that facilitate protein–lipid interactions (51, 60). To look for an effect in Rce1, the activity of purified Rce1 in GDN detergent was measured in the presence of lipids that represent components of the ER membrane (61). Phosphatidylcholine (PC), phosphatidylethanolamine (PE), phosphatidylserine (PS), phosphatidic acid (PA), or brain total lipid extract, which contains a mixture of lipids, were added to purified Rce1 and the activity was assessed using the standard assay. These lipids were tested using a molar ratio of 50:1 relative to Rce1, corresponding to concentrations of 15 μM lipid and 300 nM Rce1. The addition of PE or PC, which are the primary lipid components of the ER membrane, increased Rce1 activity by approximately 1.6- and 2.5-fold, respectively (Fig. 4*A*). PS had no significant effect. On the other hand, PA inhibited Rce1 activity, reducing it to only ∼ 14% of the activity observed in detergent alone.

**Figure 4:**
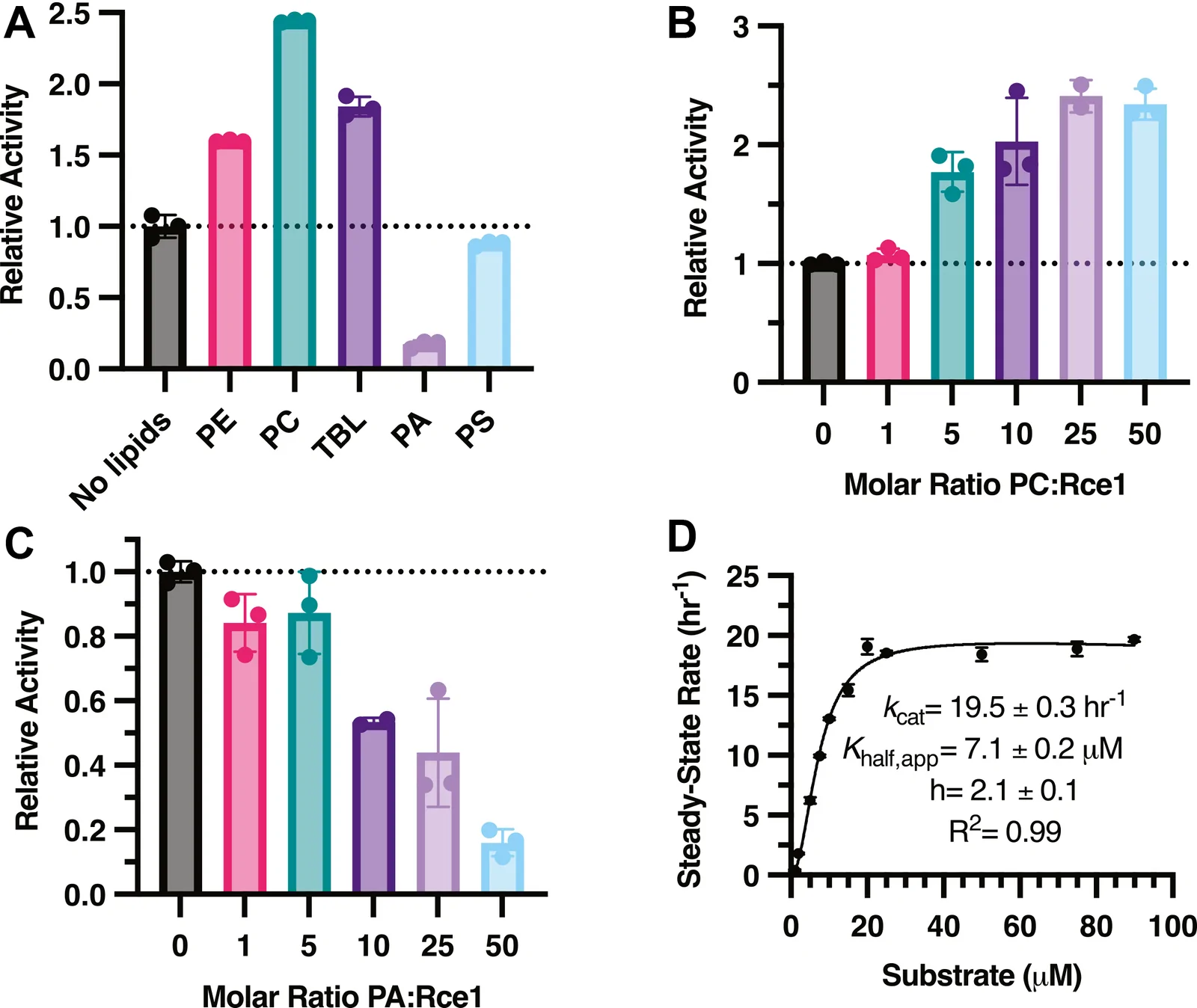
**Rce1 activity and kinetics are altered by lipids.***A*, relative activity of purified Rce1 in the presence of the indicated lipids at a 50:1 M ratio to Rce1. *B* and *C*, titrations of the PC and PA lipids at the indicated molar ratios. *D*, catalytic activity of purified Rce1 in the presence of 25:1 M ratio of PC, shown as a plot of the steady-state rate as a function of substrate (farnesylated peptide) concentration. Data are fit to an allosteric model (Experimental Procedures). Derived parameters are indicated with the S.E. of the fit. n = 3 for all experiments in this figure. PA, phosphatidic acid; PC, phosphatidylcholine; Rce1, RAS-converting enzyme, TBL, total brain lipid.

To further probe the effects of lipids, a range of concentrations of the stimulatory lipid PC and the inhibitory lipid PA were tested. Both lipids showed dose-dependent responses (Fig. 4, *B* and *C*). For PC, the half-maximal activation effect was observed with an approximately 5:1 M ratio of PC to Rce1. For PA, the half-maximal inhibition effect occurred with an approximately 10:1 M ratio. PC is the most abundant lipid in the ER membrane, representing approximately 50% of the total lipid composition, suggesting that any effect on the enzyme would be constitutive in a cellular setting (61, 62). On the other hand, PA comprises a relatively small fraction (between 0.5–7%). The concentration of PA in the ER membrane is thought to vary and dynamically regulate cellular processes (63), which raises the possibility that this lipid could modulate the activity of Rce1 in cells. However, as these experiments are unable to differentiate between the effects of direct lipid interactions or indirect effects such as changes in micellar properties or substrate partitioning, the mechanistic basis of the lipid effects is unclear.

Having identified that the ER-abundant lipid PC increases the activity of purified Rce1, we sought to study the kinetics of the enzyme in the presence of PC. Figure 4*D* shows a plot of substrate concentration *versus* enzymatic rate for purified Rce1 supplemented with a 25:1 M ratio of PC (0.3 μM Rce1 and 7.5 μM PC). Strikingly, the enzyme is no longer inhibited at high concentrations of substrate. The data display sigmoidal kinetics, in which the rate rises rapidly in the lower concentration range, with a Hill coefficient of approximately 2, a *k*_cat_ of 19.5 ± 0.3 h^−1^, and a *K*_half,app_ of 7.1 ± 0.2 μM. While the data for microsomes were consistent with Michaelis-Menten kinetics, the microsome data also fit well to a sigmoidal model using a Hill coefficient of 2 (the R^2^ values of the fits are 0.93 and 0.92, respectively). As observed for the sample in GDN alone, the purified protein in GDN-PC displays a higher apparent affinity for the substrate than Rce1 in microsomal membranes, possibly due to the relative surface area effect. We find that the GDN-PC mixture elevates *k*_cat_ to the level recorded in microsomal membranes.

### Pre-steady-state kinetics identify product release as the rate limiting step

Pre-steady-state kinetic analysis can reveal mechanistic insights beyond what is obtainable from steady-state analysis, such as the rates of steps within a reaction coordinate (64). Pre-steady-state kinetic experiments typically require specialized equipment because most enzymes turn over more rapidly than common experimental techniques can resolve (65). However, because Rce1 turns over on the order of minutes, we reasoned that pre-steady-state events might be detectable by adjusting our standard experimental procedure to observe early events. First, we reduced the amount of time between initiation of the reaction (mixing of enzyme and substrate) and subsequent reading of the fluorescence from approximately 1 min to approximately 10 s. Second, we increased the frequency of fluorescence measurements during the assay to one measurement every 2 s. Finally, we increased the concentration of Rce1 in the assay from 0.3 μM to 0.9 μM or 1.8 μM to generate higher signal for product formed. With these modifications and using a saturating (80 μM) concentration of substrate considerably greater than *K*_half,app_, we observed that product formation increased rapidly during the first minute of fluorescence recordings, consistent with a pre-steady-state burst (64, 66), before slowing and continuing in a linear steady-state manner (Fig. 5*A*). A burst phase was more apparent using 0.9 and 1.8 μM enzyme concentrations due to higher signal than the 0.3 μM condition (Fig. 5*A*). The data were fit to a kinetic model that considers both the burst phase and the steady-state phase, consisting of a single exponential and a linear component (Experimental Procedures). The linear phase reveals steady-state *k*_cat_ values of ∼ 20 h^−1^ with 0.3 μM and 0.9 μM enzyme concentrations, consistent with the rate constant determined using the standard steady-state assay conditions. A slightly lower *k*_cat_ (∼12 h^−1^) was observed at the 1.8 μM enzyme concentration, which could be due to the large amount of PC lipids used to reach the 25:1 M ratio. Fitting the data to the kinetic model yields a rate constant for the burst phase (*k*_obs_) of approximately 200 h^−1^, which is approximately 10-fold higher than the steady-state rate constant. The data also reveal the burst amplitude, which corresponds to the y-intercept of the linear phase. The burst amplitude was difficult to discern from experiments using 0.3 μM enzyme concentration, whereas 0.9 μM and 1.8 μM enzyme concentrations indicated amplitudes of 0.17 μM and 0.45 μM, respectively. The burst amplitude provides information regarding the amount of enzyme in the reaction that is catalytically active, indicating that at least 19% to 25% of Rce1 is active (obtained by dividing the burst amplitude by the total enzyme concentration). The observed amplitudes provide a lower bound on the fraction of catalytically active enzyme, both because we are blinded to the initial ∼10 s of the reaction and because they equal the active enzyme concentration only when *k*_obs_ >> *k*_cat_.

**Figure 5:**
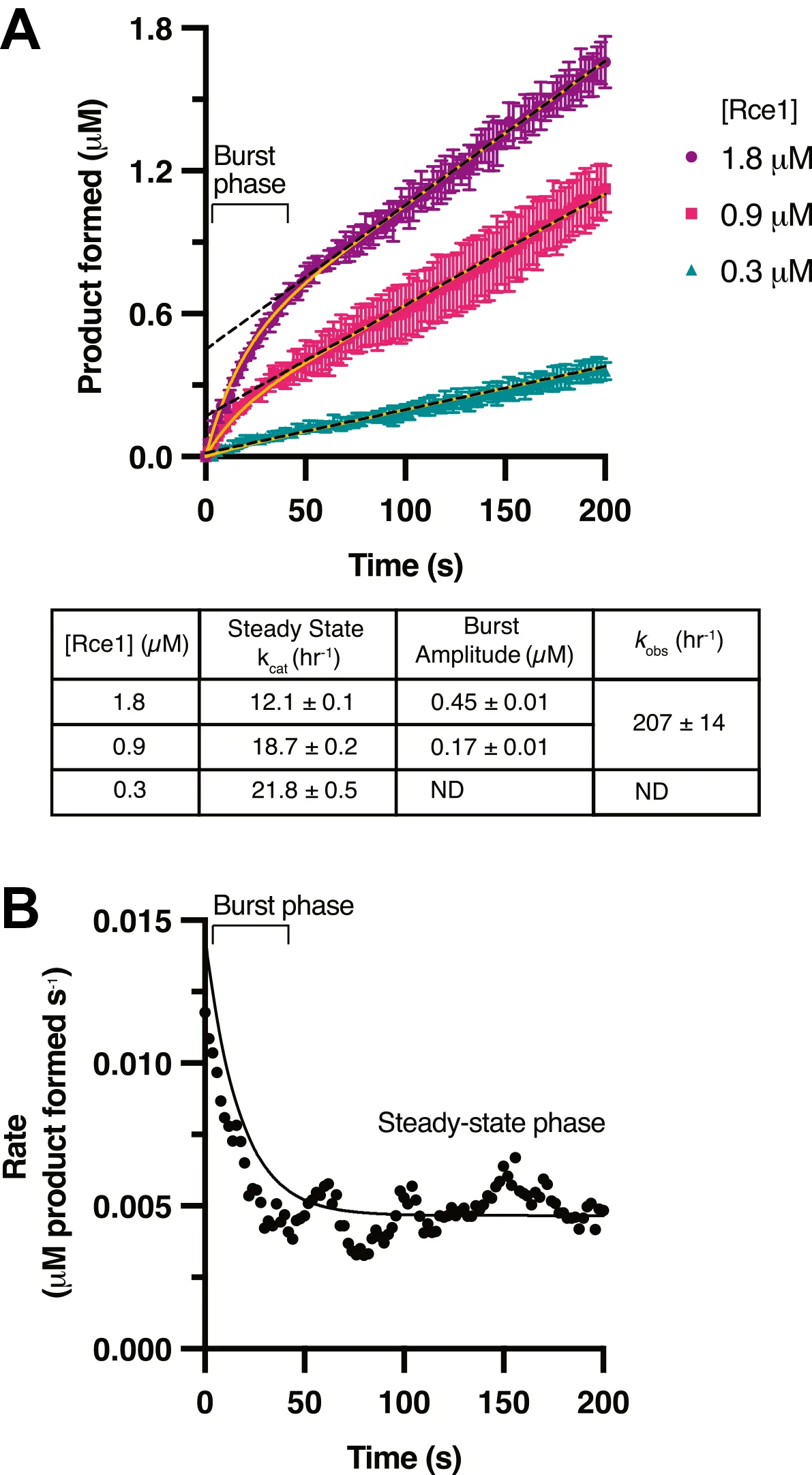
**Pre-steady-state kinetics of Rce1.***A*, product formed *versus* time using purified Rce1 (in GDN with PC) at the indicated enzyme concentrations, with saturating substrate (80 μM farnesylated peptide). Data were fit to a pre-steady-state burst equation (*gold lines*) to calculate kinetic parameters. *Dashed lines* represent linear (steady-state) regions and were extrapolated to the y-axis to obtain burst amplitudes. In the plot, time and amount of product formed represent values following the first fluorescence reading, which is approximately 10 s after initiation of the reaction. The burst amplitude and *k*_obs_ were not determined (ND) for the 0.3 μM Rce1 condition due to low signal. *B*, plot of the rate of product formation as a function of time (from the 0.9 μM Rce1 condition), calculated from the slope in (*A*) at each data point. The *solid line* represents the derivative of the pre-steady-state burst equation with respect to time. GDN, glyco-diosgenin; PC, phosphatidylcholine; Rce1, RAS-converting enzyme.

The observation of a pre-steady-state burst is indicative of a mechanism where a fast initial step is followed by a slower, rate-limiting step. The assay detects the separation of the fluorophore and its quencher following hydrolysis of the peptide bond. There are two products of the reaction, the proteolyzed prenylated peptide (now lacking the “aaX” residues) and the “aaX” residues. Release of either product from the enzyme would cause an increase in fluorescence. The burst rate constant, *k*_obs_, therefore reflects the fast events preceding the rate-limiting step. Because these pre-steady-state measurements were performed at a single, saturating substrate concentration, we report *k*_obs_ as an observed rate constant and do not resolve it into the microscopic rate constants for substrate binding, cleavage, and release of the first product. Our data indicate that the release of one of these products is the rate-limiting step in the reaction cycle, representing the *k*_cat_ rate constant. We suspect that the release of the proteolyzed prenylated product, due to its hydrophobic nature and prenyl anchor, would be slower than release of the “aaX” peptide. If this is the case, *k*_cat_ would represent the rate constant for release of the proteolyzed prenylated product.

### Rce1 is inhibited by Zn^2+^ and other divalent cations but not by common protease inhibitors

Numerous classes of covalent and non-covalent protease inhibitors are available for water soluble proteases. Many of these inhibitors are mechanism based, such that the inhibitor-sensitivity profile of an enzyme can give insight into the chemical mechanism. For example, proteases that use catalytic cysteine, serine, or aspartic amino acids are often sensitive to distinct classes of inhibitors (67). We evaluated the sensitivity of purified Rce1 to a cocktail containing a broad spectrum of protease inhibitors. No inhibition was observed when these compounds were included in the activity assay (Fig. 6*A*). This result agrees with previous analyses of yeast and human Rce1 in microsomes, which were also insensitive to common protease inhibitors (9, 33, 34), and supports the hypothesis that Rce1 uses a mechanism distinct from water-soluble proteases. However, some of the tested compounds may not have access to the catalytic site, owing to its presumed hydrophobic nature, which accommodates the prenyl group of Rce1 substrates.

**Figure 6:**
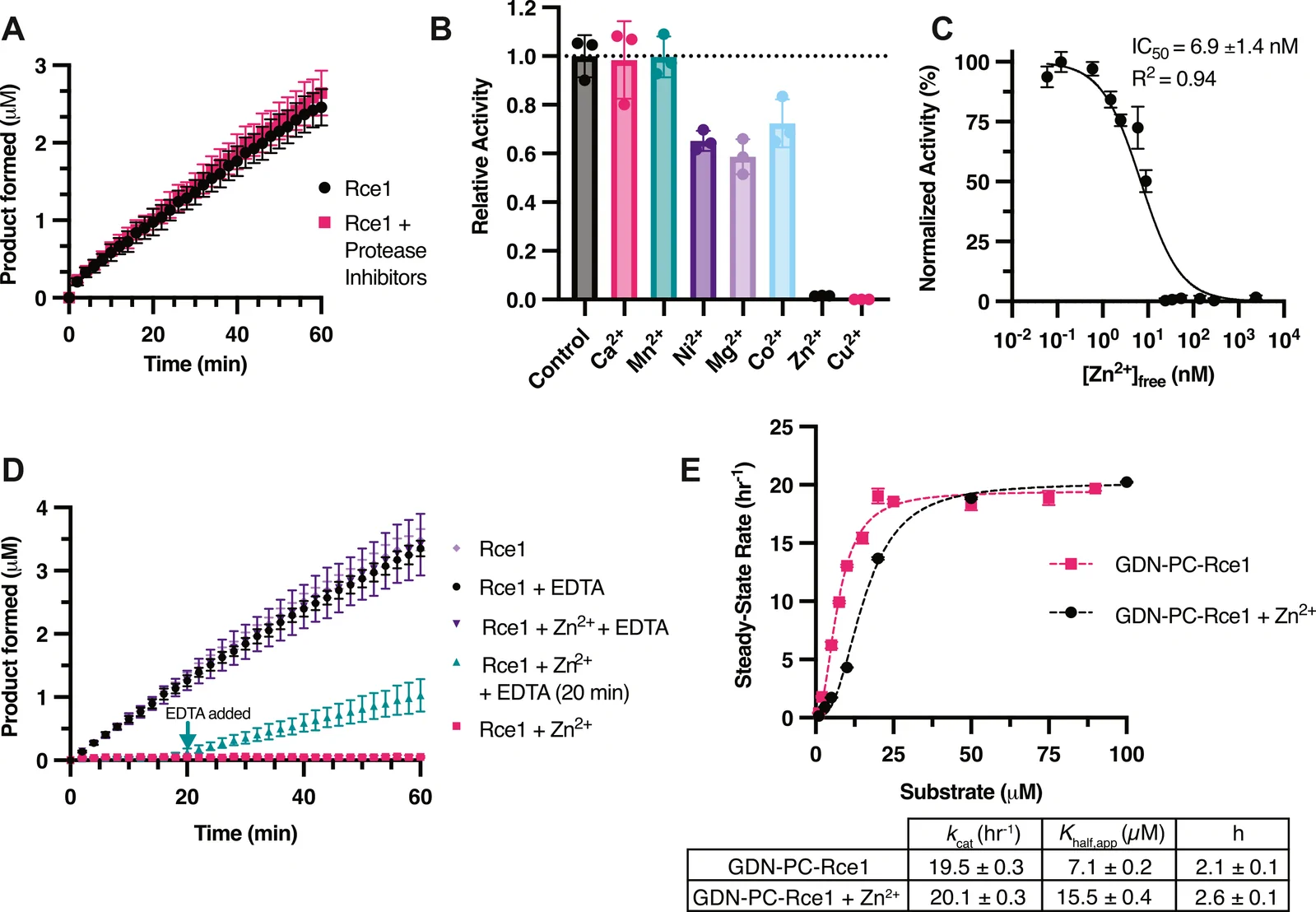
**Potent and reversible inhibition of Rce1 by Zn^2+^ but not general protease inhibitors.***A*, Rce1 is insensitive to broad-spectrum protease inhibitors targeting canonical serine, cysteine, and aspartic proteases. The standard assay is shown in the presence and absence of these inhibitors (which include AEBSF, aprotinin, bestatin, E−64, leupeptin, and pepstatin). *B*, Rce1 is inhibited by Cu^2+^ and Zn^2+^, but not the other divalent cations tested. Activity of GDN-purified Rce1 using the standard assay supplemented with 100 μM of the indicated divalent cations relative to without added metal ion (control). *C*, Rce1 is potently inhibited by Zn^2+^. The relative activity of GDN-purified Rce1 is shown in the presence of the indicated concentrations of [Zn^2+^]_free_, which were obtained using a Zn-NTA buffering system (Experimental Procedures). A *solid line* represents a fit to a normalized inhibition curve, with derived parameters indicated with the standard error of the fit. *D*, inhibition by Zn^2+^ is reversible. Standard product formed-*versus*-time plots are shown for Rce1 treated in various ways. Data labeled “Rce1” represents the standard assay condition. “Rce1 + EDTA” indicates the inclusion of 5 mM EDTA in the assay buffer. “Rce1 + Zn^2+^” indicates the inclusion of 100 μM ZnCl_2_ in the assay buffer and the resulting inhibition of enzyme activity. “Rce1 + Zn^2+^ + EDTA” indicates the inclusion of both 100 μM ZnCl_2_ and 5 mM EDTA in the assay buffer, which reveals full enzymatic activity. “Rce1 + Zn^2+^ + EDTA (20 min)” indicates the inclusion of 100 μM ZnCl_2_ in the assay buffer, which inhibits the enzyme, followed by the addition of 5 mM EDTA during the assay (*arrow*), which relieves inhibition. *E*, catalytic activity of purified Rce1 in the presence of 25:1 M ratio of PC with and without ∼50 nM [Zn^2+^]_free_, shown as a plot of the steady-state rate as a function of substrate concentration. This concentration of [Zn^2+^]_free_ inhibited the GDN-PC-Rce1 enzyme condition by approximately 50% when using 10 μM substrate. Data are fit to an allosteric sigmoidal model, with derived parameters indicated with the S.E. of the fit. n = 3 for all experiments in this figure; some error bars are smaller than data points. GDN, glyco-diosgenin; PC, phosphatidylcholine; Rce1, RAS-converting enzyme.

While Rce1 is insensitive to common protease inhibitors, it has been observed previously that microsomal preparations of the yeast and human enzymes are inhibited by micromolar amounts of Zn^2+^ (9, 29, 37). We assessed the effects of divalent cations on the activity of purified *Dm*Rce1 by including 100 μM concentrations of Ca^2+^, Mg^2+^, Mn^2+^, Ni^2+^, Co^2+^, Zn^2+^, or Cu^2+^ in the standard assay (Fig. 6*B*). Ca^2+^ and Mn^2+^ had no discernible effect, while Ni^2+^, Mg^2+^, and Co^2+^ inhibited the enzyme slightly. Enzymatic activity was completely inhibited by 100 μM Zn^2+^ or Cu^2+^.

The free concentration of Cu^2+^ is exceedingly low in eukaryotic cells, which suggests its observed inhibitory effect on Rce1 is not biologically relevant (68, 69). On the other hand, the free concentration of Zn^2+^ ([Zn^2+^]_free_) is dynamically regulated in the picomolar to nanomolar range and is thought to modulate the activity of certain enzymes (70, 71). Because 100 μM Zn^2+^ completely inhibited Rce1, we sought to investigate the effects of lower concentrations. Our activity assay required a relatively high (300 nM) concentration of Rce1 to obtain robust fluorescence signals. Therefore, a Zn-NTA buffering system was used to obtain low concentrations of [Zn^2+^]_free_. This buffering system provided a reservoir of zinc such that [Zn^2+^]_free_ lower than the concentration of enzyme could be evaluated. Testing a range of [Zn^2+^]_free_ revealed an IC_50_ of approximately 7 nM for GDN-purified Rce1 (Fig. 6*C*). An affinity in the physiological range raises the possibility that Rce1 is regulated by Zn^2+^ *in vivo*. Consistent with dynamic regulation, we found that inhibition by Zn^2+^ is reversible. Inhibition by 100 μM Zn^2+^ is completely alleviated by the subsequent addition of the divalent chelator EDTA, which otherwise has no effect on enzyme activity (Fig. 6*D*).

We wondered whether Zn^2+^ might act as a competitive inhibitor with respect to the prenylated-CaaX substrate. To evaluate this, we partially inhibited purified GDN-PC-Rce1 with a modest concentration of [Zn^2+^]_free_, and analyzed the steady-state rate *versus* substrate concentration relationship. We observed a substantial increase in the *K*_half,app_ from 7.1 ± 0.2 μM without Zn^2+^ to 15.5 ± 0.4 μM with it, but the *k*_cat_ was analogous (Fig. 6*E*). The kinetic profile, characterized by a markedly increased *K*_half,app_ with an essentially unchanged *k*_cat_, indicates competitive inhibition (64). We conclude that Zn^2+^ is a competitive inhibitor with respect to the prenylated-CaaX substrate.

### Rce1 is not a metalloprotease

We sought to determine the metal content of the purified protein to further investigate whether Zn^2+^ or another metal may have a catalytic role. Analysis by inductively coupled plasma mass spectrometry (ICP-MS) revealed substoichiometric amounts of zinc, copper, and iron (Fig. 7*A*). A metal with a catalytic role would be expected to be present in stoichiometric amounts, but we considered the possibility that only the metal-bound fraction of the preparation is catalytically active. To test this, we analyzed protein that had been transiently treated with the chelating agent EDTA. ICP-MS revealed substantially reduced quantities of these metals in the EDTA-treated sample, yet EDTA treatment had no effect on catalytic activity (Figs. 6*D* and 7*B*). Because activity is retained when metal content is depleted well below stoichiometric levels, we conclude that Rce1 does not require a metal ion for catalysis.

**Figure 7:**
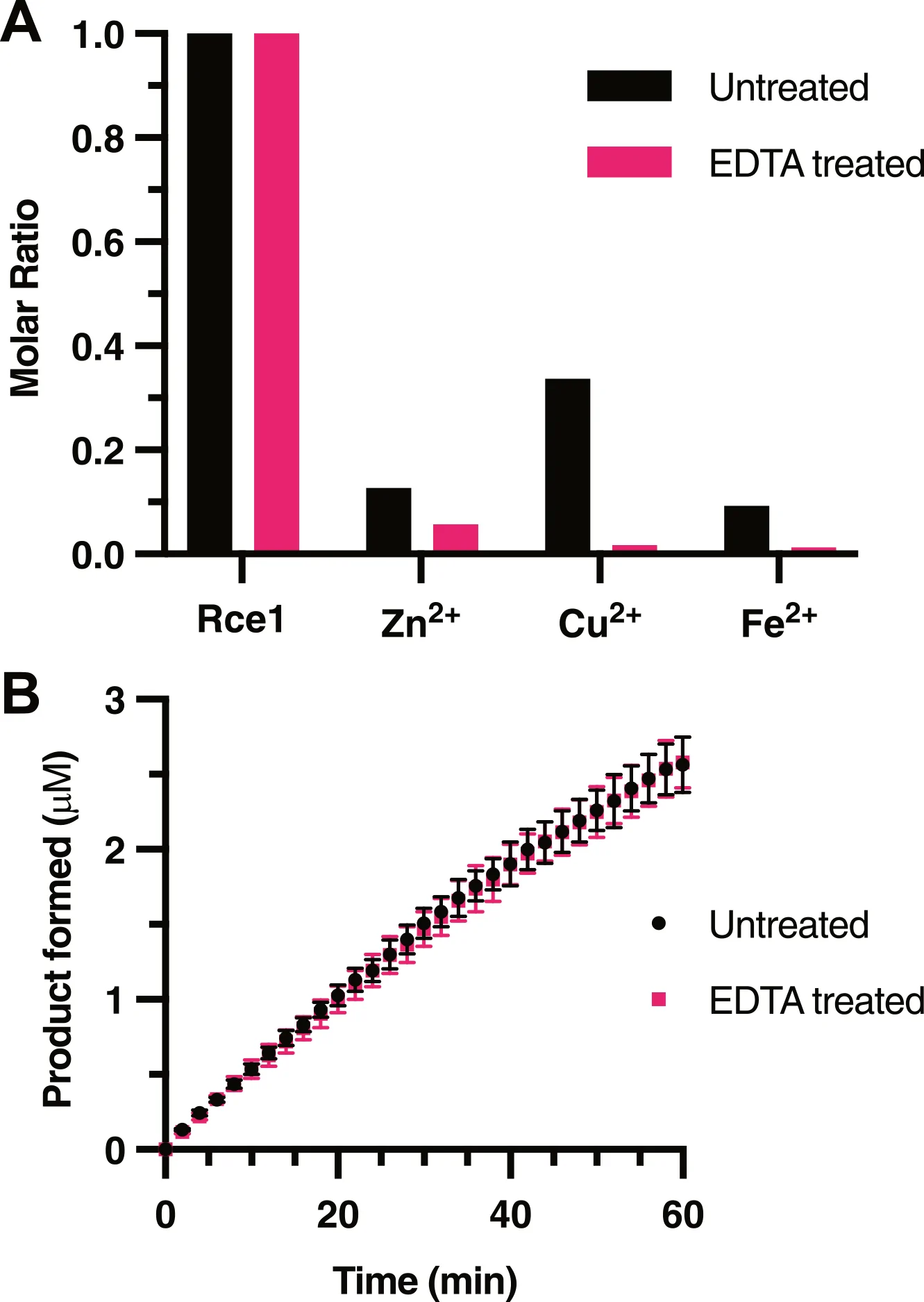
**Rce1 is not a metalloenzyme.***A*, the metal content of purified Rce1. Purified Rce1 and purified Rce1 that had been transiently treated with 5 mM EDTA were analyzed for metal content using ICP-MS. Molar ratios of the indicated metals are shown relative to Rce1. In the sample that was not treated with EDTA, three metals (Zn^2+^, Cu^2+^ and Fe^2+^) showed concentrations markedly above buffer controls. However, transient treatment of the purified enzyme with EDTA (and subsequent removal of EDTA) substantially reduced the concentrations of these metals but had no effect on catalytic activity, as shown in (*B*). Full data for the ICP-MS analysis are shown in Table S1. *B*, aliquots of samples that were used for ICP-MS were analyzed for catalytic activity under standard conditions, showing that the reductions in Zn^2+^, Cu^2+^ and Fe^2+^ by EDTA treatment does not alter enzyme activity. ICP-MS, inductively coupled plasma mass spectrometry; Rce1, RAS-converting enzyme.

## Discussion

In this study, we purified and studied the catalytic properties of *Dm*Rce1. The purified protein recapitulates known properties of eukaryotic Rce1 enzymes that had been investigated in nonpurified settings, which include the requirement for a lipid moiety attached to the cysteine residue of the CaaX motif for proteolytic cleavage. Purification of the protein enabled investigation of the kinetic properties of the enzyme. We find that enzymatic activity depends on the identity of the detergent—the protein has undetectable activity in the detergent TDM but is highly active in the detergent GDN. Because this detergent-dependence is reversible (the protein is first exposed to TDM and exchanged into GDN), we suspect the effect is due to the nature of the detergent micelle, which is markedly larger for TDM and could potentially impede substrate access and/or product release.

The catalytic activity of GDN-purified Rce1 displays sigmoidal dependence on the substrate concentration at low substrate concentrations and substrate inhibition at high concentrations. We found that the activity of the purified enzyme was inhibited by the lipid PA and stimulated by the highly abundant ER lipid PC. Rce1 also displayed sigmoidal kinetics (Hill coefficient ∼ 2) in the presence of PC, but the addition of this lipid eliminated the inhibitory effect of high concentrations of substrate. From a fit of the substrate-versus-rate relationship of this GDN-PC-Rce1 sample, we determined the enzyme’s kinetic parameters: *k*_cat_ of 19.5 ± 0.3 h^−1^ and *K*_half,app_ of 7.1 ± 0.2 μM. As observed for other integral membrane proteases (51, 52, 53, 54), *k*_cat_ of Rce1 is much lower than what is typical for water-soluble proteases. By quantifying the amount of Rce1 in unpurified microsomal membrane preparations, we also evaluated the kinetic properties of the enzyme in cell membranes. This analysis revealed a similar *k*_cat_ of 20.0 ± 3.2 h^−1^. The substrate-versus-rate relationship for Rce1 in these membranes can be fit with either a Michaelis-Menten or a sigmoidal kinetic (Hill coefficient = 2) model, raising the possibility that Rce1 may also exhibit sigmoidal kinetics in cell membranes. Rce1 in these membranes had lower apparent affinity for the substrate (*e.g. K*_M,app_ of 56 ± 16 μM) in comparison to the purified protein. As has been proposed for other membrane enzymes (52), this reduced apparent affinity may be due to the larger surface area of a membrane relative to a detergent micelle and the effective local concentration of Rce1 substrates, which partition into the membrane.

The activities of intramembrane proteins are often affected by lipids directly through binding, or indirectly through changes in properties of the lipid bilayer, such as charge, fluidity, or thickness (51, 56, 57, 58, 59). For example, PARL, a small rhomboid protease localized to the inner mitochondrial membrane, has a two-to ten-fold increase in activity when reconstituted into proteoliposomes containing the inner mitochondrial lipid, cardiolipin, compared to proteoliposomes lacking this lipid (51). Similarly, the activity of the integral membrane protease β-secretase depends on the lipid composition (56). We observed various effects for Rce1 in mixed detergent-lipid micelles. The lipids PC and PE, which are abundant in the ER membrane, representing approximately 50% and 20% of its lipid content, stimulated the activity of GDN-purified Rce1. On the other hand, the lipid PA, which is less abundant (estimated between 0.5% and 7%), reduced the activity of GDN-purified Rce1 (61, 62). The levels of PA in the ER vary depending on cellular conditions (62, 63). PA acts as a dynamic signaling molecule or ligand of other membrane proteins, including PIEZO ion channels and Patched receptors (72, 73). These considerations raise the hypothesis that the activity of Rce1 could be modulated *in vivo* by PA. It is worth noting that the effects we observed were using mixed detergent-lipid micelles. It is possible that the lipid effects could result from changes in the properties of the micelle rather than from specific interactions with the enzyme. The effects of lipids on the activity of the enzyme in lipid membranes require further study.

By assessing the metal content of the purified protein, we show that Rce1 does not require metal ions for catalytic activity, thereby demonstrating that Rce1 is not a metalloenzyme. While metals do not have a role in catalysis, we found that purified *Dm*Rce1 is inhibited by Zn^2+^, which is in agreement with previous studies of human and yeast Rce1 showing that ∼100 μM concentrations of Zn^2+^ completely inhibit enzymatic activity (9, 29, 37). Using a buffering system, we titrated [Zn^2+^]_free_ and observed an IC_50_ of approximately 7 nM, which is close to the estimated free concentration in eukaryotic cells (70). Other proteases are thought to be dynamically regulated by Zn^2+^ (70, 71). For instance, the apoptotic caspases-3, -6, and -8, which have comparable inhibition constants (*K*_i_ of 7 nM, 3 nM, and 4 nM, respectively), are thought to be regulated by Zn^2+^ *in vivo* (74). Because it is responsible for processing all prenylated CaaX proteins in cells, Rce1 had generally been assumed to be constitutively active (9). However, recent studies indicate that the activity of Rce1 can be modulated by the deubiquitinating enzyme USP17 (75). Our studies raise the possibility that its enzymatic activity may also be affected by changes in the cellular [Zn^2+^]_free_. Because Rce1 occupies an obligatory, nonredundant step in CaaX processing, such regulation—whether by Zn^2+^, lipids, USP17, or other factors—could influence the flux of substrates through the prenylation-proteolysis-methylation pathway.

Our data indicate that Zn^2+^ is a competitive inhibitor with respect to substrate. Previous studies suggest that E175, H208, and H261, types of amino acids often associated with Zn^2+^ binding, are located in the active site (9, 29, 36, 37, 38). Structural predictions using AlphaFold of human and *Drosophila* Rce1 enzymes reveal the potential locations of these amino acids (Fig. 8, *A* and *B*). In these models, E175, H208, and H261 form a triad in which their side chains are proximal to one another. The structural arrangement of this Glu-His-His triad is reminiscent of arrangements of similar triads that are known to coordinate Zn^2+^ in other proteins. For example, the integral membrane protease ZMPSTE24, which has no discernible primary sequence similarity to Rce1, contains a linear sequence motif, HEXXH, within an α-helix that creates a His-Glu-His triad that coordinates Zn^2+^ (Fig. 8*C*) (30, 76, 77, 78). In Rce1, while each of the amino acids of the Glu-His-His triad originates from a different region of secondary structure, rather than a single α-helix, the three-dimensional positioning of these amino acids in the predicted structure approximately corresponds with the arrangement found in ZMPSTE24 (and other HEXXH Zn^2+^-binding motifs) (Fig. 8*B*). We hypothesize that this is the site of Zn^2+^-mediated inhibition of Rce1 and that Zn^2+^ inhibits the enzyme by directly coordinating residues that would otherwise be involved in catalysis. Consistent with this hypothesis, when a zinc ion is included in an AlphaFold prediction for Rce1, it is placed at the Glu-His-His triad (Fig. 8*D*). Interestingly, while Rce1 and ZMPSTE24 are both integral membrane proteases, the active site Zn^2+^ serves a catalytic role in ZMPSTE24 while a metal ion is not involved in Rce1 catalysis and Zn^2+^ acts as an inhibitor.

**Figure 8:**
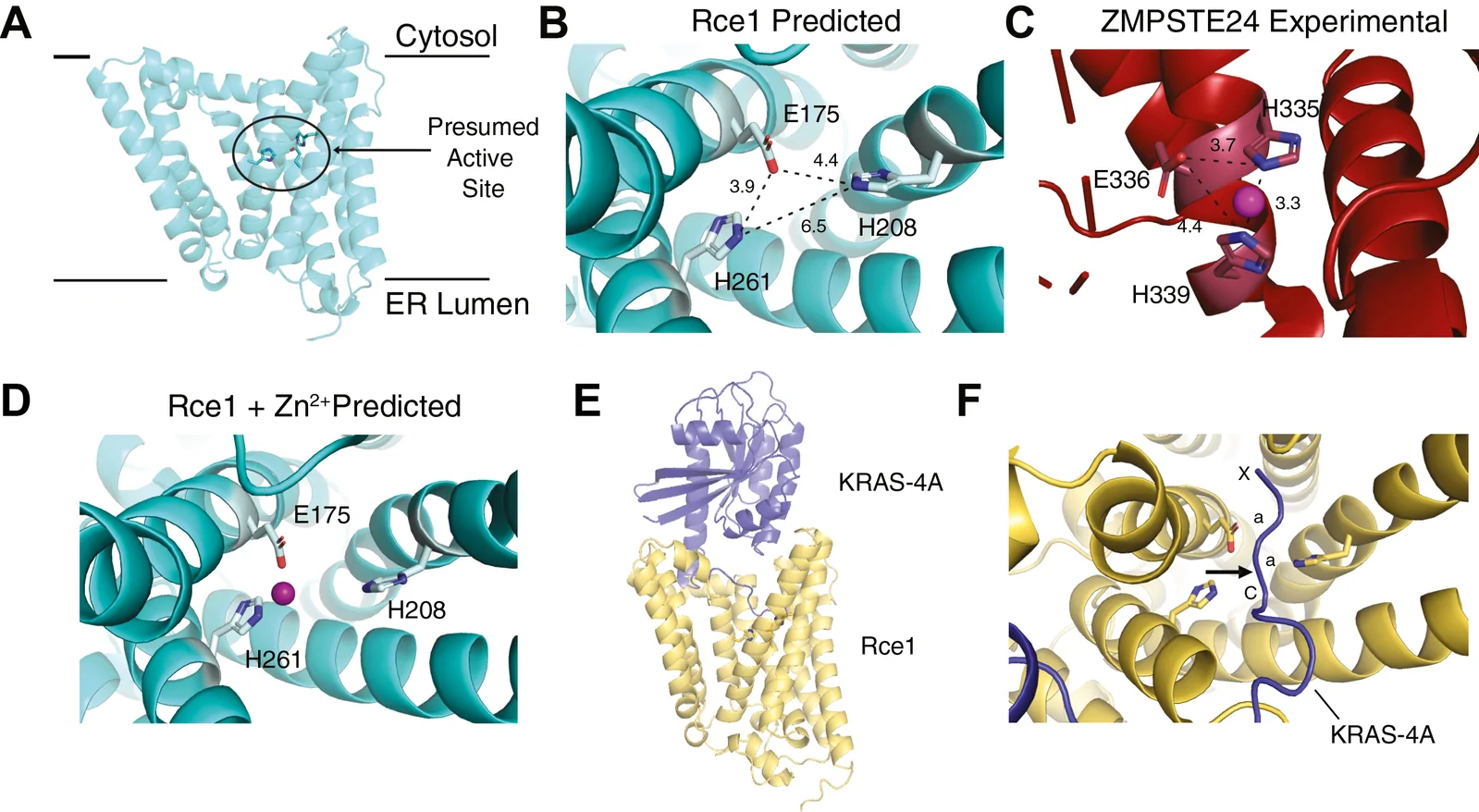
**Predicted structures of Rce1 inform hypotheses for Zn^2+^ inhibition and substrate binding.***A*, AlphaFold3 predicted model of DmRce1, showing the location of E175, H208, and H261 (human Rce1 numbering) in the presumed active site. The model is shown in cartoon representation, with *horizontal lines* indicating approximate boundaries of a lipid membrane. *B*, close-up view of the predicted active site. The arrangement of E175, H208, and H261 (*sticks*) is shown with the distances (in Å) between the amino acid side chains as *dashed lines*. *C*, the active site of human ZMPSTE24 revealed by its X-ray structure (PDB: 5SYT) suggests similarities regarding possible Zn^2+^ binding in Rce1. In ZMPSTE24, a catalytic Zn^2+^ (*sphere*) is coordinated by one glutamate and two histidine residues (*sticks*) that have similar spatial arrangements as E175, H208, and H261 in the predicted structure of Rce1. In ZMPSTE24, the Zn^2+^-coordinating residues are found on a singular α-helix as part of a HEXXH motif, whereas in Rce1, they are contributed by three different α-helices. *D*, possible inhibitory Zn^2+^ site in Rce1. When prompted to include Zn^2+^ in the prediction, AlphaFold3 places the ion (*sphere*) in the presumed active site, where it is surrounded by E175, H208, and H261. *E* and *F*, ColabFold prediction of a complex between *Hs*Rce1 (*yellow cartoon*) and KRAS-4A (*purple cartoon*). Overall and active-site views are shown in (*E*) and (*F*), respectively. The CaaX region of KRAS-4A, even though it does not contain an isoprenoid in the prediction, is positioned in the active site adjacent to E175, H208, and H261. An *arrow* represents the scissile bond of the reaction. PDB, Protein Data Bank; Rce1, RAS-converting enzyme.

Large-scale computational proteomics using ColabFold has been used to predict biologically relevant protein–protein interactions based on predicted structural complexes (79, 80, 81, 82). When querying Rce1, RAS proteins appear as highly confident interacting partners, even though an isoprenoid is not included in the prediction algorithm (Table S2). In a predicted complex structure between Rce1 and KRAS-4A from ColabFold, the CaaX motif of KRAS-4A is placed in the presumed active site of Rce1, with the scissile bond adjacent to the Glu-His-His triad in approximately the same location as the predicted inhibitory Zn^2+^ site (Fig. 8, *E* and *F*). The overlap of the predicted substrate and Zn^2+^ sites agrees with our data indicating that Zn^2+^ is a competitive inhibitor of Rce1 with respect to the prenylated CaaX substrate. This Zn^2+^-based mechanism of competitive inhibition would be an elegant means of dynamic regulation *in vivo*, and it might be exploited for development of Rce1-specific inhibitors with potential utility for cancer.

The low *k*_cat_ of Rce1 (approximately 20 h^−1^) facilitated studying the reaction using pre-steady-state kinetics. By making such measurements, we observed a distinct burst phase, revealing a pre-steady-state rate constant (*k*_obs_) of approximately 200 h^−1^. Because the fluorescence signal arises from separation of the fluorophore and quencher—that is, from release of a cleavage product rather than from bond hydrolysis itself—the burst reports a fast product-release event, and the rate-limiting step of the turnover reaction (*k*_cat_) is a subsequent, slower release of one of the two products (the prenylated product or the ‘aaX’ residues). Interestingly, release of the prenylated product is the rate-limiting step for prenylation by FTase and likely GGTase-I, where dissociation of the prenylated product limits turnover (14, 83, 84). Because of its hydrophobic prenyl anchor, we suspect that release of the proteolyzed prenylated product, rather than the aaX peptide, represents the rate-limiting step of Rce1.

The availability of a purified, catalytically active Rce1 removes a barrier to studying the biochemical properties of this enzyme and provides a tractable system for further mechanistic, structural, and inhibitor-discovery efforts. High-resolution structures of Rce1 in complex with substrates and products would be beneficial to define the enzyme’s unusual, metal-independent proteolytic mechanism and to guide the design of selective inhibitors.

## Experimental procedures

### Expression and purification of *Dm*Rce1

*Dm*Rce1 (Uniprot accession number: Q9U1H8) was selected from among approximately twenty eukaryotic Rce1 orthologs as a candidate for purification based on expression level and monodisperse elution profile in detergent-containing solutions using FSEC of protein that was produced in HEK-293 cells (85). For large scale preparation, cDNA encoding *Dm*Rce1, spanning amino acids 13 to 302, was cloned into the EcoRI and SalI sites of the *P. pastoris* pPICZ-A vector (Invitrogen) with a C-terminal GFP tag, which was separated by a linker containing a PreScission protease cleavage site. Transformation into *P. pastoris*, cell culture, and cryogenic mechanical lysis followed previously described methods (86). Lysed cells were stored at −80 °C until use.

The purification of *Dm*Rce1 was performed as follows. A total of 14 g of lysed cell powder were resuspended in 140 ml lysis buffer [100 mM Hepes, pH 7.5, 150 mM NaCl, 0.1 mg/ml DNAse (Sigma-Aldrich), 1:1000 Protease inhibitor cocktail set III (Sigma-Aldrich), 0.5 mM AEBSF (Gold Biotechnology)] and stirred at 600 rpm for 10 min at RT, adjusting the pH to 7.5 using 1 M NaOH. At this point, 1.5 g of n-tetradecyl-β-D-maltopyranoside (TDM, Anatrace) detergent was added and the solution was stirred at 600 rpm for 60 min. The sample was then centrifuged at 50,000*g* for 1 h at 4 °C to separate solubilized protein from the insoluble fraction, followed by filtration of the supernatant using a 0.22 μm bottletop filter (Millipore). The filtered sample was incubated with 3 ml of GFP-nanobody coupled to CNBr-activated Sepharose resin (GE Healthcare) for 2 h at 4 °C while rotating end over end (87). The beads were washed on a gravity-flow column using 100 ml buffer [20 mM Hepes, pH 7.5, 150 mM NaCl, 0.2 mM glyco-diosgenin (GDN, Anatrace)] at 4 °C to promote detergent exchange and remove contaminants. Washed beads were then incubated with 3 ml cutting buffer (20 mM Hepes, pH 7.5, 150 mM NaCl, 0.2 mM GDN, 1 mM DTT, and 0.2 mg PreScission protease) overnight at 4 °C. After collecting the flow through containing *Dm*Rce1, the column was washed with 3 ml of additional buffer (20 mM Hepes, pH 7.5, 150 mM NaCl, and 0.1 mM GDN), the fractions were combined, and the sample was concentrated with a 100-kDa MWCO spin concentrator (Amicon, 4 ml, 4 °C) to approximately 0.4 ml. The protein was then further purified using SEC (GE Heathcare, Superose 6 Increase 10/300 GL column) in buffer (20 mM Hepes, pH 7.5, 150 mM NaCl, and 0.1 mM GDN). The fractions corresponding to *Dm*Rce1 were collected and either used immediately for enzymatic assays, or concentrated to greater than 2 mg/ml, flash frozen in liquid nitrogen, and stored at −80 °C. Freshly prepared and previously frozen protein displayed indistinguishable enzymatic activity. Protein concentration was determined from the absorption of 280 nm light and the theoretical extinction coefficient of ε = 60,390 M^−1^ cm^−1^ based upon the amino acid sequence. For protein exclusively in TDM (Fig. 2), 0.5 mM TDM was used throughout purification.

For the preparation of microsomes containing unpurified *Dm*Rce1, lysed cell powder was suspended in detergent-free buffer [100 mM Hepes, pH 7.5, 150 mM NaCl, and 0.1 mg/ml DNAse (Sigma-Aldrich), 1:1000 Protease inhibitor cocktail set III (Sigma-Aldrich), 0.5 mM AEBSF (Gold Biotechnology)] and mechanically agitated by stirring at 500 rpm for 5 min at RT. The pH of the solution was adjusted to between 7.5 and 8.0 using NaOH. Membranes from 12 g of lysed cell powder were then harvested by centrifugation at 43,000*g* at 4 °C for 1 h and resuspended in 5 ml buffer (20 mM Hepes, pH 7.5, 150 mM NaCl). The amount of *Dm*Rce1 protein contained within the membranes was determined from the fluorescence of GFP in the *Dm*Rce1-GFP fusion protein, analogous to previously described methods (49). Briefly, a sample of membranes was solubilized using buffer containing TDM detergent (100 mM Hepes, pH 7.5, 150 mM NaCl, 0.1 mg/ml DNAse, 1:1000 Protease inhibitor cocktail set III, 0.5 mM AEBSF, and 20 mM TDM) and run on FSEC in buffer (20 mM Hepes, pH 7.5, 150 mM NaCl, and 0.5 mM TDM). The area under the FSEC peak was compared with known quantities of purified GFP.

### Activity assay

The activity assay was based upon previously published methods for microsomal preparations of *Hs*Rce1 (47, 48) and adapted for purified protein in detergent. Briefly, a custom 9-mer peptide mimicking the C terminus of KRAS-4B (Abz-KSKTKC_f_Q_L_IM) was synthesized commercially (AnaSpec). This peptide contains a farnesylated cysteine residue (C_f_), an N-terminal Abz fluorophore, and a dinitrophenyl lysine (Q_L_) in the a_1_ position. In the absence of cleavage, Q_L_ quenches the fluorescence of Abz, and negligible fluorescence is detected. An analogous peptide without the farnesyl group was synthesized (Abz-KSKTKCQ_L_IM), as were peptides that represent the products of the reaction (Abz-KSKTKC_f_ and Q_L_IM). Known amounts of the product peptides were used to quantify the amount of product formed by Rce1. Lyophilized peptides were dissolved in DMSO to obtain stock solutions at 20 mg/ml.

In the assay, purified Rce1 (from a 300 μM stock in SEC buffer containing 0.1 mM GDN or 0.5 mM TDM) was diluted to yield a final concentration of 300 nM Rce1 in 80 μl of assay buffer (20 mM Hepes, pH 7.5, 150 mM NaCl), added to a 96-well black-bottomed plate (Thermo Fisher Scientific, Cat. No. 137101), and incubated at RT for 10 min. Reactions were initiated by adding 20 μl of peptide substrate (which had been diluted in assay buffer) to yield final concentrations between 0.1 μM and 100 μM, and the plate was inserted into a SpectraMax M5e fluorometer (Molecular Devices, running SoftMax Pro 7 software) at 25 °C. Unless noted, the standard reaction condition contained 20 μM peptide and 300 nM Rce1 purified in GDN, at pH 7.5 and 25 °C. For steady-state measurements, fluorescence readings were taken every 0.5 to 2 min for up to 3 h using wavelengths of 320 nm and 420 nm for excitation and emission, respectively. For the standard (steady-state) experiments, there is a delay of approximately 1 min between initiation of the reaction and measuring of the first fluorescent reading. Data were analyzed and visualized using GraphPad Prism 10. The synthetic peptides representing the products of the reaction were run in parallel using concentrations between 1 μM and 50 μM to create a standard curve for the conversion of relative fluorescence units to product concentration (Fig. S3). A plot of product formed *versus* time reveals an approximately linear relationship for the first 30 min, from which the steady-state catalytic rate (*k*_cat_) is determined by linear regression (using GraphPad Prism 10). For experiments in which the pH was varied, the following pH-buffers were used in place of Hepes, pH 7.5, with otherwise standard assay conditions: glycine, pH 10.5; glycine, pH 9.5; Tris, pH 8.5; MES, pH 6.5; sodium cacodylate, pH 5.5; sodium acetate, pH 4.0. To account for the possibility that the fluorescence of the proteolyzed product is partially affected by pH, relative activities at the different pH values were normalized by measuring the fluorescence of the product peptide standards at each pH value, which varied minimally (Fig. S4).

For assays with lipid additives, the following lipids were used (Avanti): 1-palmitoyl-2-oleoyl-sn-glycero-3-phosphocholine (PC, Cat. No. 850457); 1-palmitoyl-2-oleoyl-sn-glycero-3-phosphoethanolamine (PE, Cat. No. 850757); 1-palmitoyl-2-oleoyl-sn-glycero-3-phospho-L-serine (PS, Cat. No. 840034); Brain Total Lipid Extract (Cat. No. 131101); 1-palmitoyl-2-oleoyl-sn-glycero-3-phosphate (PA, Cat. No. 238051). The lipids were obtained as chloroform solutions, were dried under argon gas in a glass vial, resuspended in pentane, dried again under argon, and left under vacuum overnight at RT (and protected from light). Dried lipids were resuspended in water to a concentration of 10 mM and sonicated using a water bath sonicator for 3 min with periodic cooling on ice to prevent heating from sonication. This was repeated until the solution became transparent or until 15 total minutes of sonication (certain lipids do not become fully transparent). The lipids were then solubilized by adding GDN powder to 17 mM and mixing end over end for 2 h at 4 °C. Lipids were included in the assay buffer (from the 10 mM lipid stock containing 17 mM GDN, and prior to the 10-min equilibration step) to yield lipid concentrations between 0.3 μM and 15 μM. PC and other lipids were not included in experiments unless otherwise noted.

The following equations were used to fit substrate concentration *versus* steady-state rate relationships (using GraphPad Prism 10). The Michaelis-Menten model (for Rce1 in microsomal membranes):

$$Y=V_{\max}X/\left(K_{M,\text{app}}+X\right)$$

An allosteric sigmoidal model (for Rce1 in GDN detergent with added PC lipid):

$$Y=V_{\max}X^{h}/\left({K_{\text{half},\text{app}}}^{h}+X^{h}\right)$$

An allosteric sigmoidal model that takes into account substrate inhibition (for Rce1 in GDN detergent without added lipids):

$$Y=V_{\max}X^{h}/\left({K_{\text{half},\text{app}}}^{h}+X^{h}\left(1+\frac{X^{h}}{K_{i}^{h}}\right)\right)$$

In these equations, *V*_max_ represents the maximal velocity of the reaction, *X* represents the substrate concentration, and *Y* represents the steady-state rate. *K*_i_ is the inhibition constant for substrate inhibition, and *h* is the Hill coefficient. The turnover number (*k*_cat_) is obtained by dividing *V*_max_ by the total enzyme concentration. *K*_half,app_ and *K*_M,app_ are the concentrations of substrate for which the enzyme reaches half maximal velocity – because these constants are dependent upon membrane/detergent partitioning, they are reported as apparent (app) values.

### Pre-steady-state analysis

Pre-steady-state analysis was performed using the activity assay described above with a saturating amount of substrate (80 μM) and with Rce1 concentrations of 0.3 μM, 0.9 μM, and 1.8 μM. The reaction mixtures contained PC lipids to yield 25:1 M ratios with respect to Rce1 (*e.g.* 7.5 μM, 22.5 μM, and 45 μM PC, respectively). In comparison to the standard steady-state experimental protocol, read times between fluorescent measurements were minimized to the shortest achievable interval (2 s) to maximize temporal resolution. The delay between initiation of the reaction (performed as described previously by the addition of peptide) and the first fluorescence reading was reduced to approximately 10 s. Because the observed burst phase lasts approximately 60 s, a meaningful portion of the burst phase occurred before fluorescence readings were collected. Fluorescence intensities were converted to the amount of product formed as described previously. Data were plotted as product formation *versus* time and were fit to a pre-steady-state burst equation (using Prism):

$$Y=A\;\left(1-e^{-k_{\text{obs}}\;*\;X}\;\right)+V_{ss}\;X$$

In this equation, *A* represents the amplitude of the pre-steady-state burst. *k*_obs_ represents the observed rate constant of the burst phase. *V*_ss_ is the steady-state velocity (linear portion of the relationship), from which *k*_cat_ can be obtained by dividing *V*_ss_ by the total enzyme concentration in the reaction. The slope shown in Figure 5*B* at each time point was calculated using a rolling linear regression with a fixed window of 10 data points, incremented by one time point at each step (to minimize noise in the slope calculations).

### Effects of divalent cations

For experiments evaluating Rce1 activity in the presence of divalent cations, the standard reaction buffer was supplemented with 100 μM of each (CaCl_2_, MnCl_2_, MgCl_2_, CoCl_2_, ZnCl_2_, and CuCl_2_). To assess the ability of Zn^2+^ to inhibit Rce1 at concentrations below the enzyme concentration used in the assay, ZnCl_2_ was buffered with nitrilotriacetic acid (NTA), as has previously been described (88). Experiments were performed using the standard assay buffer supplemented with 1 mM NTA and 10 μM to 1 mM ZnCl_2_ to obtain the desired [Zn^2+^]_free_ between 60 pM and 2.3 μM, which was calculated using MaxChelator (https://somapp.ucdmc.ucdavis.edu/pharmacology/bers/maxchelator/webmaxc/webmaxcS.htm). Rce1 activity at each [Zn^2+^]_free_ was compared to the maximum activity in the presence of 1 mM NTA without ZnCl_2_ to generate relative activities. The dose-response data were fit using nonlinear regression in GraphPad Prism 10 to the following equation.

$$Y=100/\left(1+\frac{X}{{IC}_{50}}\right)$$

In this equation, Y represents the relative activity, X is the concentration of [Zn^2+^]_free_, and IC_50_ is the value at which 50% relative activity is observed.

To assess competitive binding, kinetic assessment of Rce1 in the presence of Zn-NTA was performed in assay buffer containing PC lipids (20 mM Hepes, pH 7.5, 150 mM NaCl, 7.5 μM PC) with approximately 50 nM [Zn^2+^]_free_ determined using MaxChelator. This concentration was selected because it inhibited the GDN-PC-Rce1 enzyme condition by approximately 50% when using 10 μM of peptide substrate. Substrate concentrations between 1 μM and 100 μM were tested and fit a sigmoidal kinetic model as described previously.

### Metal analysis

To assess metal content, the purified protein was analyzed using ICP-MS at the Center for Applied Isotope Studies at the University of Georgia. The protein sample for ICP-MS analysis was prepared in 0.2 mM GDN as described, except that a reduced concentration of NaCl (75 mM) was used in the SEC buffer to limit the ICP-MS signal from NaCl. Following SEC, the protein was concentrated to approximately 250 μl at 3 mg/ml using a 100-kDa MWCO spin concentrator (Amicon), flash frozen, and stored at −80 °C. The protein sample along with an SEC buffer control, which was collected from buffer that had been run through the SEC column to account for possible metal contamination caused by SEC, were shipped on dry ice to the facility for analysis. For the EDTA-treated sample, the protein was incubated with 5 mM EDTA for 30 min at 4 °C prior to SEC. EDTA was then removed from the sample using SEC. Nitric acid was used by the Center for Applied Isotope Studies at the University of Georgia to digest the samples prior to elemental analysis.

### Model prediction with AlphaFold3

Predicted structures of *Dm*Rce1 and *Hs*Rce1 were generated using https://alphafoldserver.com/ using the primary amino acid sequences of the proteins (46). Five models were generated of each, and a representative predicted model was chosen for use in alignments (Fig. S6). Modeling of *Dm*Rce1 with Zn^2+^ was performed in the same manner by including a Zn^2+^ ion in the prediction. The zinc ion was predicted to occupy the same site in all five models. Models were viewed and aligned using PyMol v.3.1.6.1 (http://www.pymol.org/pymol). A predicted structural interaction between *Hs*Rce1 and KRAS-4A was obtained from the predictomes.org database (accession number Q9Y256, P01116; Table S2), which utilizes the ColabFold version of AlphaFold-Multimer to screen and score predicted binary protein–protein interactions (79, 80, 81, 82).

## Data availability

All data are contained within the article.

## Supporting information

This article contains supporting information.

## Conflict of interest

The authors declare that they have no conflicts of interest with the contents of this article.
