# Supplementary material for: Catalytic properties of the integral membrane RAS protease Rce1
Source: J Biol Chem. 2026 Sep 8;302(11):113525. doi: 10.1016/j.jbc.2026.113525 (PMC13734883; doi:10.1016/j.jbc.2026.113525)
Supplement: Supplementary file 1 [file mmc1.pdf]

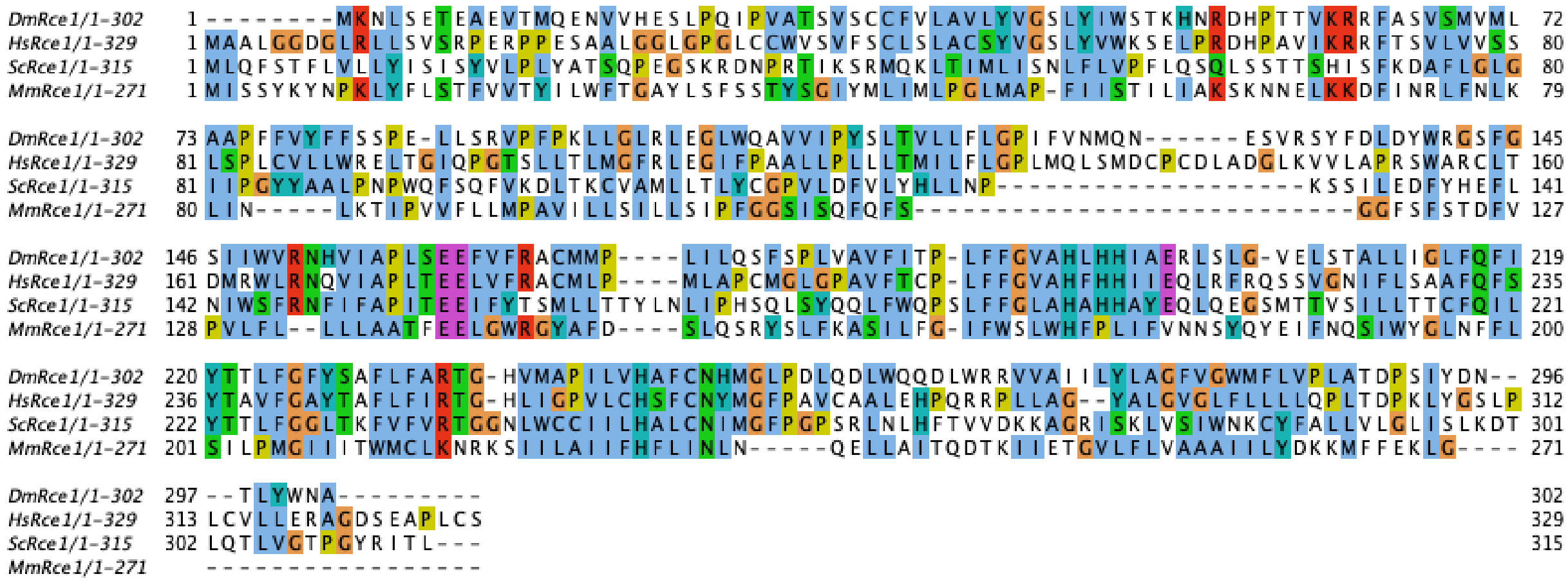

Figure S1. **Sequence alignments of indicated Rce1 orthologs.** Sequence alignment of *Dm*Rce1 (Uniprot accession number: Q9U1H8), *Hs*Rce1 (Q9Y256), *Sc*Rce1 (Q03530), and the archaeal protein *Mm*Rce1 (Q6LZY8). *Dm*Rce1 shares 38% sequence identity with *Hs*Rce1 and 30% sequence identity with *Sc*Rce1. Alignment and coloring were made with Clustal.

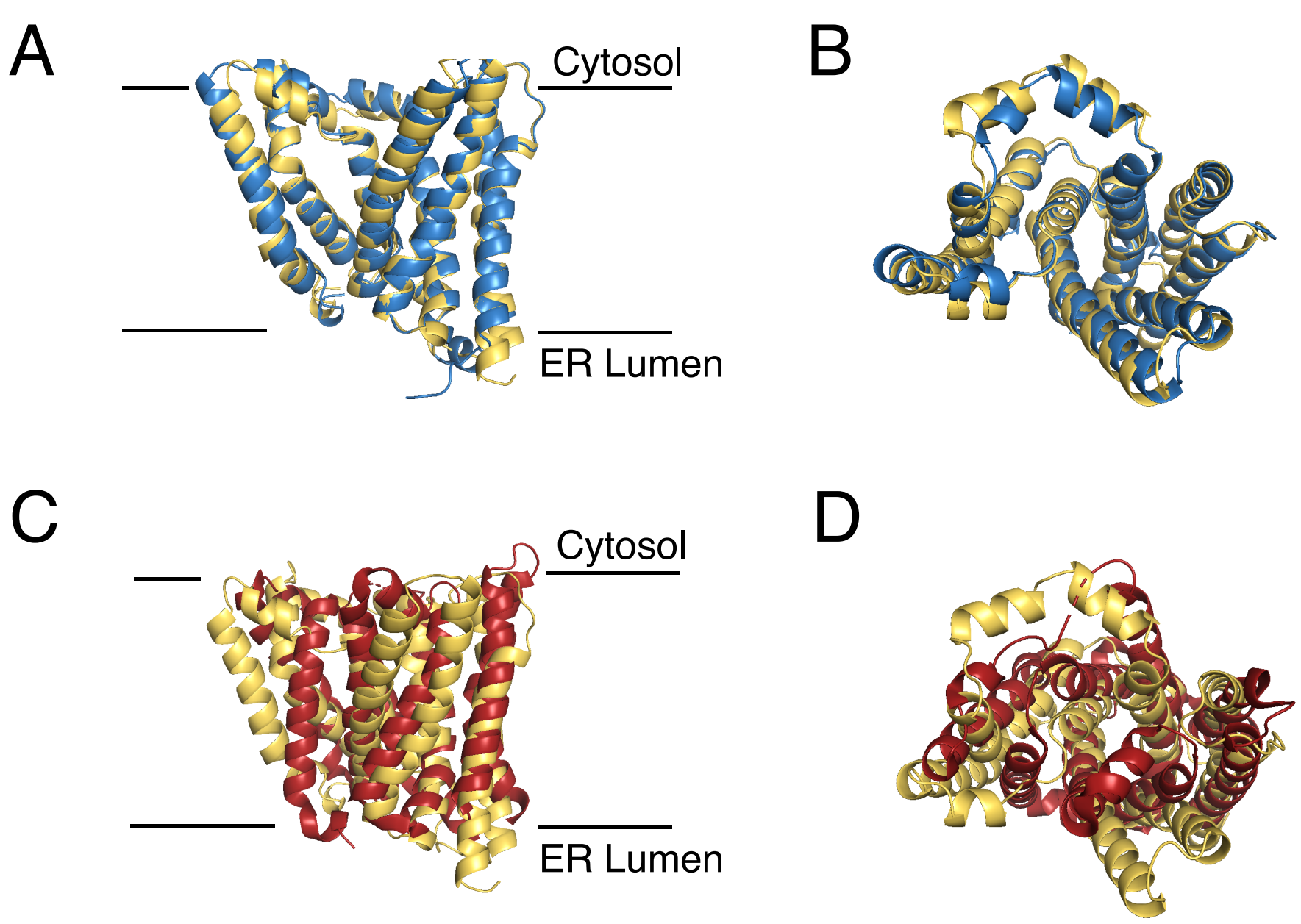

Figure S2. **Structural alignments of predicted *Hs*Rce1, predicted *Dm*Rce1, and the X-ray structure of *Mm*Rce1.** A and B, Side and top view of *Dm*Rce1 (Cyan) aligned with *Hs*Rce1 (Gold) (RMSD=0.69 Å for Cα positions). Structures were predicted using AlphaFold3, generating 5 predicted models from the primary amino acid sequences (Figure S6). A representative structure of each was chosen for alignment. C and D, Side and top view of *Mm*Rce1 (PDB ID: 4CAD; red) aligned with representative predicted structure of *Hs*Rce1 (gold) (RMSD=6.8 Å).

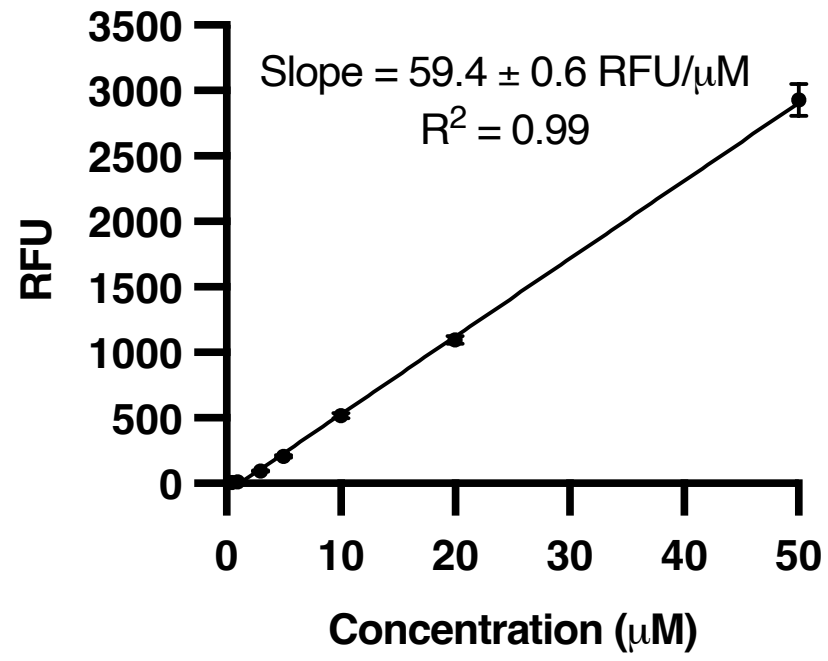

Figure S3. **Representative calibration curve using fluorescent product peptide.** Fluorescence signal from the product peptides (Abz-KSKTKC$_f$ and Q$_L$IM) plotted as a function of concentration (0, 1 µM, 2 µM, 5 µM, 10 µM, 20 µM, and 50 µM). The solid line represents a linear fit of the data, which was used to convert the RFU observed in enzymatic assays to the concentration of product formed. An analogous calibration was done each time an assay was performed (n=3, with error bars representing SEM). Derived slope is indicated with the S.E. of the fit.

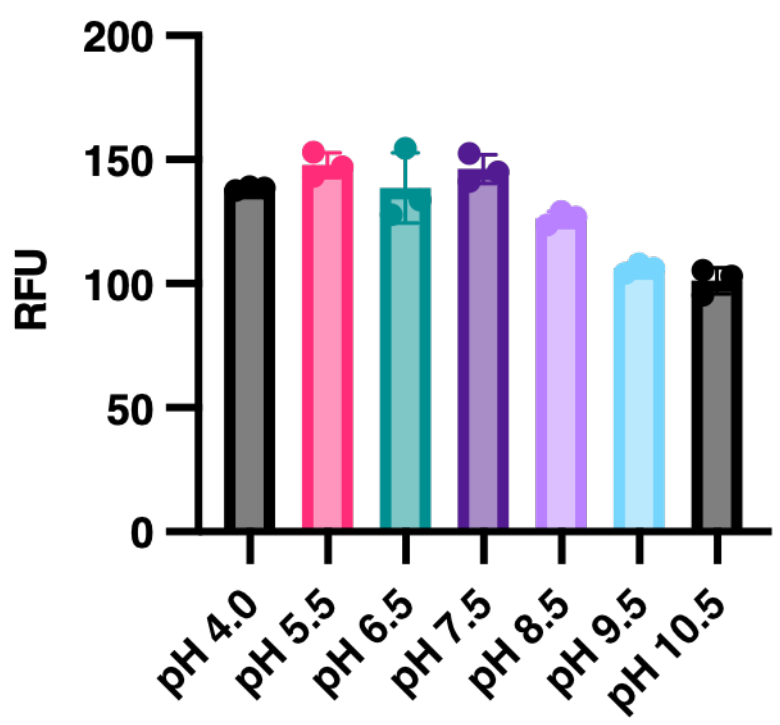

Figure S4. **Effects of pH on standards.** The RFU of the product peptide (Abz-KSKTKC$_f$) standard was measured at 5 µM between pH 4.0 and pH 10.5 to determine the effect of pH on fluorescence signal (n=3). Values from 1 hour were taken and normalized to the value at pH 7.5 to create a standard adjusted fluorescence that accounts for fluorescence differences of the product peptide across pH in plots of Rce1 activity at various pH values.

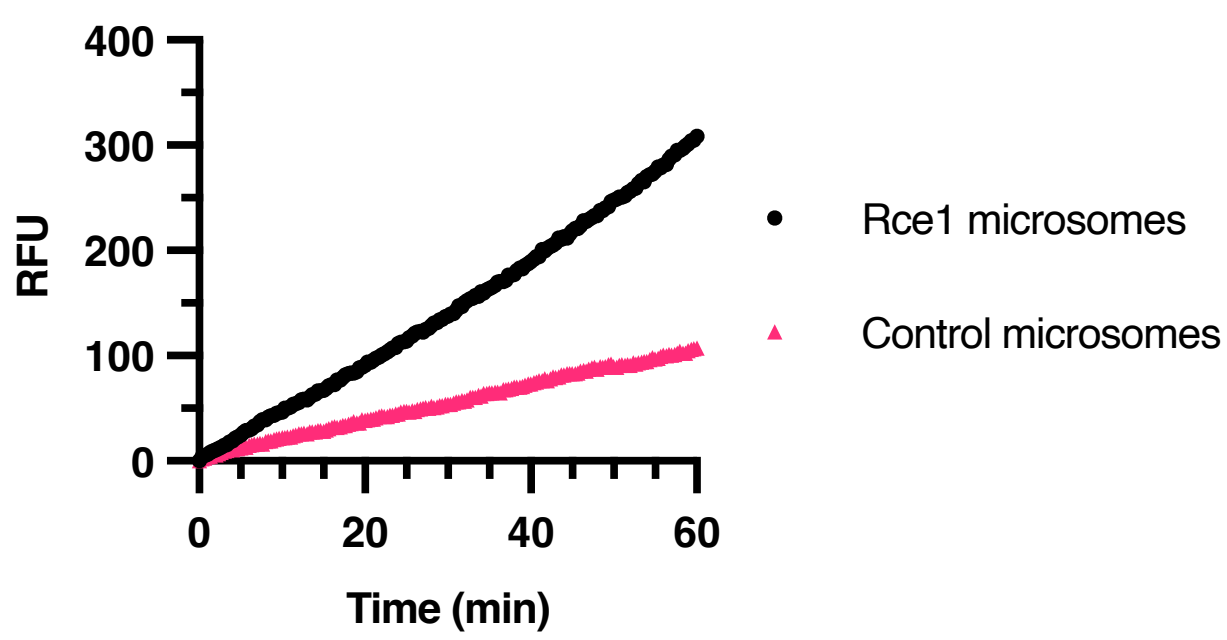

Figure S5. **Background subtraction for Rce1 microsomes.** Microsomes from untransformed yeast contain enzymes that hydrolyze the prenylated CaaX substrate (red trace). This background activity was subtracted from *Dm*Rce1 microsomes (black trace). Linear regression of the first 30 minutes was used to determine steady-state rate.

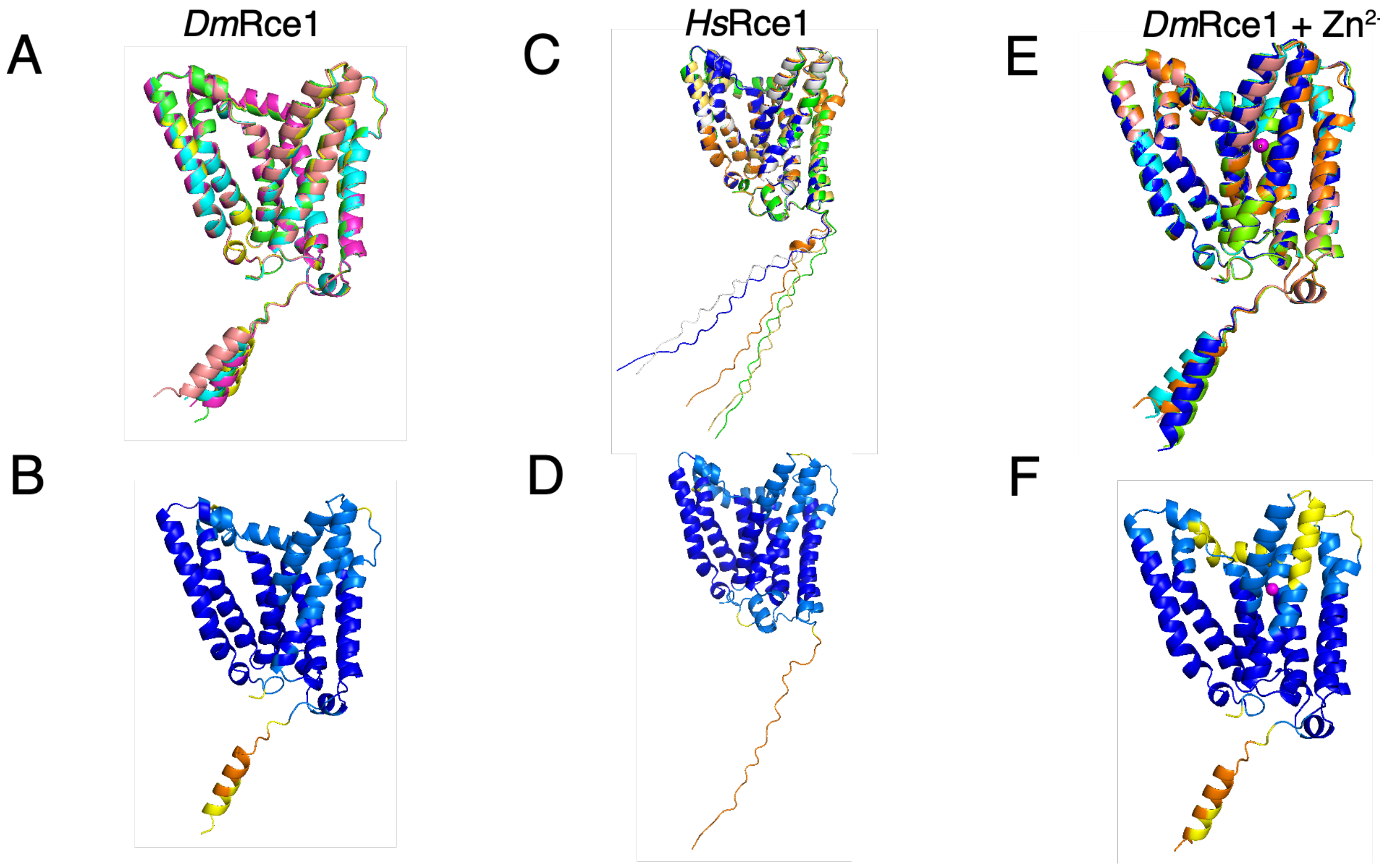

Figure S6. **Structural prediction of *Dm*Rce1, *Hs*Rce1, and *Dm*Rce1 with Zn^{2+} using AlphaFold.** A, C, E, Overlays of five predicted structures for *Dm*Rce1 (Uniprot accession number: Q9U1H8), *Hs*Rce1 (Uniprot accession number: Q9Y256), and *Dm*Rce1 with Zn^{2+} demonstrating a high degree of uniformity in each model prediction. Each model is in a different color. B, D, F, Representative predicted models of Rce1 from (A, C, and E) colored by estimated model accuracy (blue; pLDDT>90), good backbone predictions (marine; pLDDT between 70 and 90), low confidence (yellow; pLDDT between 50 and 70), and very low confidence (orange; pLDDT below 50). Zn^{2+} (E and F) is shown as a magenta sphere and occupies the same position in all five models of *Dm*Rce1 with Zn^{2+}.

	Untreated Rce1			EDTA-treated Rce1		
Metal (µg/kg)	Protein sample	Buffer	Molar Ratio to Rce1	Protein sample	Buffer	Molar Ratio to Rce1
Li	BQ<194	BQ<184	N/A	BQ<70.2	BQ<78.4	N/A
Be	BQ<7.5	BQ<7.12	N/A	1.73	0.596	0.00125
Na	1.52E+06	1.62E+06	N/A	1.71E+06	1.65E+06	N/A
Mg	BQ<13.9	BQ<13.2	N/A	BQ<42.1	BQ<47.0	N/A
Al	61.9*	BQ<60.5	0.023*	BQ<99.2	BQ<111	N/A
K	BQ<2470	BQ<2345	N/A	BQ<436	BQ<488	N/A
V	BQ<2.99	BQ<2.84	N/A	BQ<2.31	BQ<2.58	N/A
Cr	BQ<2.91	BQ<2.76	N/A	BQ<3.40	BQ<3.80	N/A
Mn	BQ<2.91	BQ<2.77	N/A	BQ<2.19	BQ<2.45	N/A
Fe	520	BQ<49.5	0.0932	127	55	0.013
Co	BQ<1.89	BQ<1.8	N/A	BQ<1.36	BQ<1.52	N/A
Ni	BQ<3.58	BQ<3.4	N/A	BQ<3.48	BQ<3.88	N/A
Cu	2157	16.8	0.337	111	BQ<3.93	0.0174
Zn	833	BQ<6.36	0.127	377	BQ<14.7	0.0574
Ga	BQ<2.44	BQ<2.32	N/A	BQ<2.49	BQ<2.78	N/A
As	BQ<2.11	BQ<2.00	N/A	BQ<3.49	BQ<3.90	N/A
Se	BQ<16.3	BQ<15.5	N/A	BQ<19.5	BQ<21.7	N/A
Rb	BQ<2.65	BQ<2.52	N/A	5.33	1.03	0.000377
Sr	BQ<2.42	BQ<2.30	N/A	1.82	BQ<1.48	0.000208
Ag	BQ<1.26	BQ<1.19	N/A	BQ<1.74	BQ<1.95	N/A

Table S1. **ICP-MS results for metal analysis of purified Rce1.** Metal concentrations (µg/kg) for purified Rce1 samples (untreated and EDTA treated) and corresponding buffer controls. The limit of quantitation was determined for each metal using blanks. Values determined to be below the limit of quantitation are reported as "BQ". Highlighted rows reflect metal detected >0.01 molar ratio relative to Rce1. *A small amount of aluminum was detected just above the detection limit in the untreated sample.

UniProt_ID	UniProt_Name	Gene_Symbol	SPOC_score	FDR_(%)	Avg_models	ipTM_max	STRING_score
P01111	RASN_HUMAN	NRAS	0.682	21.2	0.83	0.7	813
P01116	RASK_HUMAN	KRAS	0.676	21.2	0.88	0.64	801
O43493	TGON2_HUMAN	TGOLN2	0.622	29.2	0.464	0.51	0
Q92731	ESR2_HUMAN	ESR2	0.604	31.8	0.625	0.28	0
Q8TCT7	SPP2B_HUMAN	SPPL2B	0.526	42.5	0.4	0.55	212
Q96RK4	BBS4_HUMAN	BBS4	0.52	44.5	0.333	0.41	167
P03372	ESR1_HUMAN	ESR1	0.48	53.6	0.417	0.25	0
P37173	TGFR2_HUMAN	TGFBR2	0.443	59.8	0.333	0.43	151
P63092	GNAS2_HUMAN	GNAS	0.336	66.4	0.357	0.24	0
A5PLL7	PDES1_HUMAN	PEDS1	0.125	70.5	0.333	0.36	0
Q92989	CLP1_HUMAN	CLP 1	0.119	70.8	0.333	0.55	0
O60725	ICMT_HUMAN	ICMT	0.081	73.1	0.333	0.37	998
Q9Y6F6	IRAG1_HUMAN	IRAG1	0.075	73.1	0.333	0.36	292
P0CG47	UBB_HUMAN	UBB	0.061	75	0.333	0.24	0
P23443	KS6B1_HUMAN	RPS6KB1	0.053	75.5	0.333	0.67	0

**O43865**	SAHH2_HUMAN	AHCYL1	0.05	75.5	0.333	0.53	0
**P50454**	SERPH_HUMAN	SERPINH1	0.043	75.9	0.338	0.4	0
**Q8IXX5**	T183A_HUMAN	TMEM183A	0.042	75.9	0.333	0.2	0
**Q9Y3E5**	PTH2_HUMAN	PTRH2	0.035	75.9	0.426	0.29	0
**Q8N3G9**	TM130_HUMAN	TMEM130	0.034	76.6	0.59	0.6	0
**Q9Y6A1**	POMT1_HUMAN	POMT1	0.034	76.6	0.578	0.5	0
**Q9BRR6**	ADPGK_HUMAN	ADPGK	0.034	76.6	0.468	0.49	0
**Q96HN2**	SAHH3_HUMAN	AHCYL2	0.024	77.7	0.333	0.36	0
**Q9NXS2**	QPCTL_HUMAN	QPCTL	0.023	77.7	0.522	0.3	0
**P80370**	DLK1_HUMAN	DLK1	0.022	77.7	0.424	0.28	0
**O00144**	FZD9_HUMAN	FZD9	0.017	77.7	0.533	0.48	0
**Q9HDC9**	APMAP_HUMAN	APMAP	0.015	77.7	0.333	0.56	0
**O14610**	GBGT2_HUMAN	GNGT2	0.013	81.5	0.333	0.28	197
**Q8NET1**	D108B_HUMAN	DEFB108B	0.01	81.5	0.381	0.3	220
**Q8NFJ5**	RAI3_HUMAN	GPRC5A	0	91.4	0	0.23	0
**Q13616**	CUL1_HUMAN	CUL1	0	91.4	0	0.19	0
**O75881**	CP7B1_HUMAN	CYP7B1	0	91.4	0	0.2	0
**Q8TC12**	RDH11_HUMAN	RDH11	0	91.4	0	0.19	0
**O15120**	PLCB_HUMAN	AGPAT2	0	91.4	0	0.17	0
**Q9H6H4**	REEP4_HUMAN	REEP4	0	91.4	0	0.14	0
**O15258**	RER1_HUMAN	RER1	0	91.4	0	0.12	0
**P49795**	RGS19_HUMAN	RGS19	0	91.4	0	0.23	0
**Q6NTF9**	RHBD2_HUMAN	RHBDD2	0	91.4	0	0.13	0
**Q8N2K0**	ABD12_HUMAN	ABHD12	0	91.4	0	0.22	0
**Q5XPI4**	RN123_HUMAN	RNF123	0	91.4	0	0.24	297
**Q8N8Q8**	COX18_HUMAN	COX18	0	91.4	0	0.16	0
**Q13948**	CASP_HUMAN	CUX1	0	91.4	0	0.23	0
**O43709**	BUD23_HUMAN	BUD23	0	91.4	0	0.2	0
**Q9UBX8**	B4GT6_HUMAN	B4GALT6	0	91.4	0	0.16	0
**Q96N21**	AP4AT_HUMAN	TEPSIN	0	91.4	0	0.44	0
**Q9BT22**	ALG1_HUMAN	ALG1	0	91.4	0.333	0.21	0
**Q96HV5**	TM41A_HUMAN	TMEM41A	0	91.4	0	0.15	0
**P57088**	TMM33_HUMAN	TMEM33	0	91.4	0	0.14	0
**Q8N2E6**	TOR2X_HUMAN	TOR2A	0	91.4	0	0.2	0
**P30536**	TSPO_HUMAN	TSPO	0	91.4	0	0.14	179
**Q9Y2B2**	PIGL_HUMAN	PIGL	0	91.4	0	0.16	0
**Q8N5M9**	JAGN1_HUMAN	JAGN1	0	91.4	0	0.2	0
**Q9Y673**	ALG5_HUMAN	ALG5	0	91.4	0	0.17	0
**Q9C073**	F117A_HUMAN	FAM117A	0	91.4	0	0.25	0
**Q9Y5Z9**	UBIA1_HUMAN	UBIAD1	0	91.4	0	0.13	326

**Q96CS3**	FAF2_HUMAN	FAF2	0	91.4	0	0.19	0
**P49327**	FAS_HUMAN	FASN	0	91.4	0	0.33	0
**Q8TF61**	FBX41_HUMAN	FBXO41	0	91.4	0	0.28	182
**Q8N6M3**	FITM2_HUMAN	FITM2	0	91.4	0	0.14	0
**P18074**	ERCC2_HUMAN	ERCC2	0	91.4	0	0.19	0
**Q8IWL3**	HSC20_HUMAN	HSCB	0	91.4	0	0.18	292
**Q9BW60**	ELOV1_HUMAN	ELOVL1	0	91.4	0	0.18	0
**Q9H496**	IFG15_HUMAN	TOR1AIP2	0	91.4	0	0.28	0
**Q99943**	PLCA_HUMAN	AGPAT1	0	91.4	0.333	0.21	0
**Q12756**	KIF1A_HUMAN	KIF1A	0	91.4	0	0.28	0
**O75427**	LRCH4_HUMAN	LRCH4	0	91.4	0.333	0.34	0
**Q93074**	MED12_HUMAN	MED12	0	91.4	0	0.25	0
**Q9H1A3**	METL9_HUMAN	METTL9	0	91.4	0.333	0.23	0
**Q96ES6**	MFSD3_HUMAN	MFSD3	0	91.4	0	0.17	0
**Q969G9**	NKD1_HUMAN	NKD1	0	91.4	0	0.29	0
**Q969F2**	NKD2_HUMAN	NKD2	0	91.4	0	0.28	0
**Q15738**	NSDHL_HUMAN	NSDHL	0	91.4	0.333	0.23	0
**O43913**	ORC5_HUMAN	ORC5	0	91.4	0	0.13	0
**Q8N138**	ORML3_HUMAN	ORMDL3	0	91.4	0	0.24	0
**Q32NB8**	PGPS1_HUMAN	PGS1	0	91.4	0	0.21	155

Table S2. **Proteins predicted to interact with HsRce1 from Predictome.** Predicted interactions were sorted by "Structural Prediction and Omics-Informed Classifier" (SPOC) score, which estimates interaction likelihood based on structural and biological features. "False discover rate" (FDR) estimates a percentage of hits at a certain SPOC rate likely to be false. "Average models" represents the fraction of predicted models that display the same interfacial contacts between the two proteins. STRING score reflects the STRING database's confidence that two proteins interact physically or functionally (scores <400 are unlikely to interact).
